# Racial disparity in pro-metastatic tumor microenvironment in treatment naïve breast cancer

**DOI:** 10.1038/s41523-025-00865-1

**Published:** 2026-01-06

**Authors:** Priyanka Parmar, Burcu Karadal-Ferrena, Suryansh Shukla, Andrew Miller, Chenxin Zhang, Cien Huang, Timothy D’Alfonso, Rachel Han, Esther Adler, Nurfiza Ladak, Paula S. Ginter, Susan Fineberg, Xianjun Ye, Mindy Ginsberg, Chedva Rosenbaum, Malka Felder, Yu Lin, Xiaoming Chen, Robert J. Eddy, Thomas E. Rohan, John S. Condeelis, Xiaonan Xue, Jesus Anampa, Joseph A. Sparano, David Entenberg, Maja H. Oktay

**Affiliations:** 1 Department of Surgery, Albert Einstein College of Medicine/Montefiore Medical Center, Bronx, NY USA; 2 Montefiore Einstein Comprehensive Cancer Center, Albert Einstein College of Medicine/Montefiore Medical Center, Bronx, NY USA; 3 Cancer Dormancy Institute, Albert Einstein College of Medicine/Montefiore Medical Center, Bronx, NY USA; 4 Department of Pathology, Albert Einstein College of Medicine/Montefiore Medical Center, Bronx, NY USA; 5 Gruss-Lipper Biophotonics Center, Albert Einstein College of Medicine/Montefiore Medical Center, Bronx, NY USA; 6 Integrated Imaging Program for Cancer Research, Albert Einstein College of Medicine/Montefiore Medical Center, Bronx, NY USA; 7 Department of Epidemiology & Population Health, Albert Einstein College of Medicine/Montefiore Medical Center, Bronx, NY USA; 8 Albert Einstein College of Medicine, Bronx, NY USA; 9 Department of Pathology, Memorial Sloan Kettering Cancer Center, New York, NY USA; 10 Laboratory Medicine and Molecular Diagnostics, Precision Diagnostics and Therapeutics Program, Sunnybrook Health Sciences Centre, Toronto, ON Canada; 11 Department of Pathology, NYU Grossman School of Medicine, New York, NY USA; 12 Department of Cell Biology, Albert Einstein College of Medicine/Montefiore Medical Center, Bronx, NY USA; 13 Department of Oncology, Albert Einstein College of Medicine/Montefiore Medical Center, Bronx, NY USA; 14 Division of Hematology/Oncology, Icahn School of Medicine at Mount Sinai, Tisch Cancer Institute, New York, NY USA

**Keywords:** Breast cancer, Cancer microenvironment, Prognostic markers

## Abstract

Black women with estrogen receptor-positive, HER2-negative (ER + /HER2-) breast cancer experience higher rates of distant recurrence and worse survival outcomes compared to White women. This may be due not only to disparities in social determinants of health, but also differences in the tumor microenvironment (TME), including TMEM (Tumor Microenvironment of Metastasis) doorway score. TMEM doorways serve as portals for cancer cell hematogenous dissemination to distant sites. While higher TMEM doorway scores have been observed in Black (compared to White) patients with residual ER + /HER2- breast cancer after neoadjuvant chemotherapy, this has not been evaluated in treatment-naïve primary breast cancers. Here, we report on a multi-institutional study to evaluate TMEM doorway score in 418 treatment-naïve archived human breast cancer samples, including 265 patients with ER + /HER2-, 102 with triple negative (TNBC), and 51 with HER2-positive breast cancer. In addition to analyzing TMEM doorway scores by race across breast cancer subtypes, we examined their association with distant recurrence and assessed whether the effect of TMEM doorway scores on recurrence differed by race. Black patients had significantly higher TMEM doorway score than White patients in the overall study population (median 29.9 vs 17.9, *p* < 0.001), in the ER + /HER2- (median 25.0 vs 16.8, *p* < 0.001) and the HER2-positive subset (median 37.2 vs 12.9, *p* = 0.003), but not in TNBC (median 36.2 vs 36.3, *p* = 0.86). Racial differences in macrophage density mirrored racial differences in the TMEM doorway score. In multivariate models including age, body mass index, tumor size, grade, lymph node status, and chemotherapy treatment, neither Black race nor TMEM doorway density was associated with a higher distant recurrence risk alone. However, there was a statistically significant interaction between race and high TMEM doorway score with respect to distant recurrence risk in ER + /HER2- patients; Black patients with high TMEM doorway score were 4.6-fold (95% CI 1.28–22.82, *p* = 0.03) and 4.2-fold (95% CI 1.17 – 18.23, *p* = 0.04) more likely to have a distant recurrence at 5-years and 10-years, respectively, while White patients with high TMEM doorway scores did not (p = 0.21, *p* = 0.11). Our study reveals racial disparities in the TME of women with ER + /HER2- breast cancer, which may play a critical role in driving disparities in breast cancer outcomes.

## Introduction

Black women with localized breast cancer have significantly higher mortality rates compared to White women, even in clinical trial populations where patients are healthier, have equal access to care, and clinical outcomes are evaluated after adjustment for clinicopathological factors and social determinants of health^1–5^. This persistent disparity suggests that biological factors beyond social determinants may be at play for Black women.

Racial disparities are particularly pronounced in estrogen receptor-positive/ERBB2-negative (ER + /HER2-) breast cancer, where Black patients treated with adjuvant chemotherapy experience worse outcomes than their White counterparts, a pattern that is less evident in ER- disease^3,4,6–8^. Our group previously reported that racial disparity in outcomes also exists in Black women with residual ER + /HER2- disease after treatment with neoadjuvant chemotherapy (NAC)^8,9^. In addition, findings from the TAILORx cohort revealed that compared to White women with ER + /HER2- breast cancer, Black women with this breast cancer type and intermediate OncotypeDX score have a significantly earlier recurrence^10^. Likewise, in the RxPONDER cohort, it was shown that the overall five-year disease-free survival rate for women with ER + /HER2- breast cancer is lowest for Black women, despite similar OncotypeDX scores^11^.

To understand this disparity, it is essential to distinguish between tumor-intrinsic biology (such as genomic drivers and proliferation markers) and the tumor microenvironment (TME), which includes host immune, stromal, and vasculature components and their interactions with tumor cells. TME factors can influence invasion, metastasis and therapeutic resistance^12^. Emerging evidence suggests that racial disparities in outcome may be rooted in tumor–host interactions within the TME. Prior work suggests that longstanding environmental exposures may shape distinct immune signatures in African American patients compared to European American, shaping TME composition, including higher macrophage and microvascular density, which contribute to metastasis^13^. These tumor–host interactions may contribute to worse metastatic outcomes in Black patients in ways that are not captured by current genomic tests.

A key TME-based mechanism in cancer cell dissemination to distant sites is the formation of TMEM (Tumor Microenvironment of Metastasis) doorways^14,15^. TMEM doorways arise through stable tri-cellular interactions between a tumor cell, a macrophage, and an endothelial cell, resulting in the creation of a trans-endothelial structure through which tumor cells can enter the bloodstream^16,17^. The details of TMEM Doorway function have been established using pre-clinical in vivo and in vitro models, as well as in vivo imaging^14,16,18,19^. Previous studies have shown that TMEM doorway score is an independent prognostic factor of distant recurrence in patients with ER + /HER2- breast cancer who received adjuvant chemotherapy or NAC^9,20,21^.

While our prior work demonstrated that Black patients with residual ER + /HER2- disease after neoadjuvant chemotherapy have higher TMEM doorway scores and macrophage levels^9^, it remains unknown whether these disparities exist at baseline in treatment-naïve breast cancer. In exploring this further, we propose a hypothesis that racial differences in pro-metastatic markers (such as TMEM doorways and macrophage density) are due to inherent racial differences in tumor biology. Although race is a social rather than a biologic construct, Black race is a surrogate for African ancestry.

In this multi-institutional study, we assessed TMEM doorway score, as well as macrophage and microvascular density in treatment-naïve breast tissues taken from a large group of patients (418 total, with 184 Black, 146 White, and 88 of other racial backgrounds) with the goal of providing insight into whether baseline differences in these pro-metastatic markers exist and are associated with Black vs White racial disparities in breast cancer outcomes, particularly in ER + /HER2- breast cancer.

## Results

### Patient characteristics

**Fig. 1:**
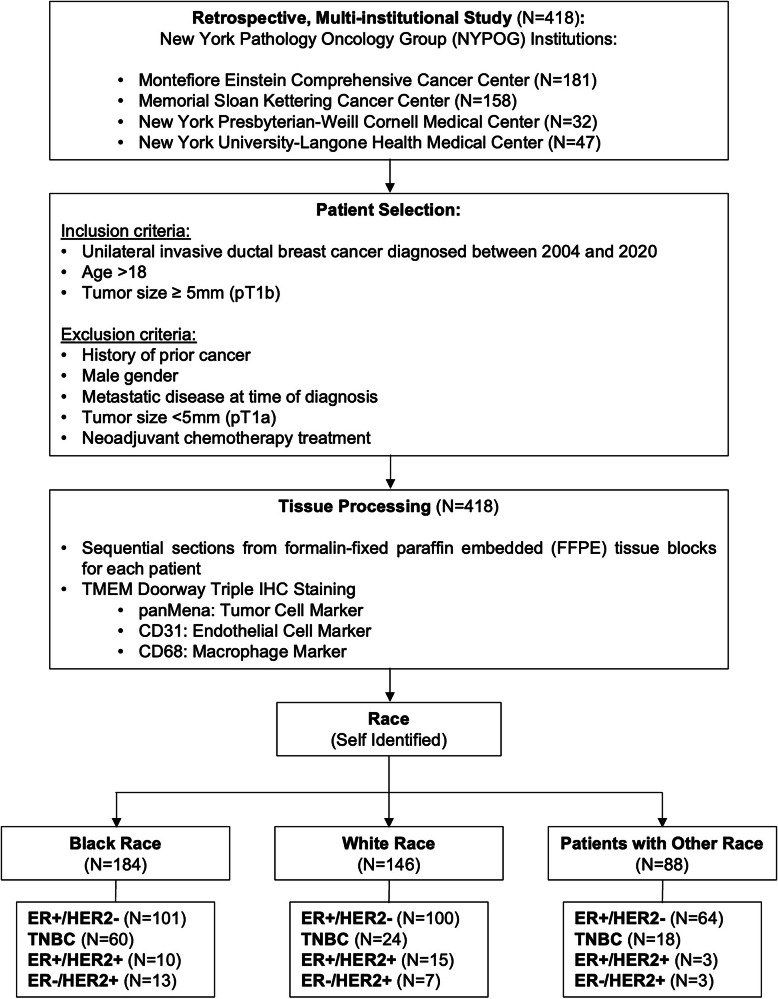
CONSORT diagram of the study.

The initial study population included 418 patients with treatment-naive invasive ductal breast carcinoma for whom formalin-fixed, paraffin-embedded tissue samples were obtained (Fig. 1). Tissue and outcome data from 184 Black, 146 White and 88 patients of other races were included in the analysis to assess differences in pro-metastatic tumor microenvironment variables. Demographics and tumor characteristics for the entire study population are presented in Table 1, stratified by (patients who developed distant recurrence) versus controls (those without clinical or radiographic evidence of recurrence). Table 2 represents data stratified by Black vs White race within each breast cancer subtype, while Table 3 represents Black vs White race across all subtypes combined. These comparisons were used to analyze outcome differences by Black and White race.

**Table 1:** Patient characteristics by cases and controls

	Cases	Controls	All Patients	*P*-value
	(*N* = 194)	(*N* = 221)	(*N* = 415)	
**Institution**				**<0.001**
Cornell	31 (16.0%)	0 (0%)	31 (7.5%)	
Montefiore	66 (34.0%)	114 (51.6%)	180 (43.4%)	
MSKCC	73 (37.6%)	84 (38.0%)	157 (37.8%)	
NYU	24 (12.4%)	23 (10.4%)	47 (11.3%)	
**Race**				0.19
White	82 (42.3%)	63 (28.5%)	145 (34.9%)	
Black	76 (39.2%)	108 (48.9%)	184 (44.3%)	
Asian	5 (2.6%)	6 (2.7%)	11 (2.7%)	
Other	31 (16.0%)	44 (19.9%)	75 (18.1%)	
**Age (years)**				0.68
Mean (SD)	56.0 (13.1)	56.8 (13.3)	56.4 (13.2)	
Median [Min, Max]	55.0 [26.0, 89.0]	56.0 [27.0, 89.0]	56.0 [26.0, 89.0]	
**Body Mass Index**				0.81
Mean (SD)	29.5 (6.66)	29.2 (6.26)	29.3 (6.43)	
Median [Min, Max]	28.2 [18.7, 56.8]	28.8 [15.4, 51.7]	28.6 [15.4, 56.8]	
Missing	33 (17.0%)	3 (1.4%)	36 (8.7%)	
**Surgery type**				0.51
BCT/Lumpectomy	107 (55.2%)	140 (63.3%)	247 (59.5%)	
Lumpectomy followed by mastectomy	8 (4.1%)	7 (3.2%)	15 (3.6%)	
Mastectomy	79 (40.7%)	72 (32.6%)	151 (36.4%)	
Missing	0 (0%)	2 (0.9%)	2 (0.5%)	
**Radiation**				0.31
Yes	127 (65.5%)	158 (71.5%)	285 (68.7%)	
No	62 (32.0%)	55 (24.9%)	117 (28.2%)	
Missing	5 (2.6%)	8 (3.6%)	13 (3.1%)	
**Tumor stage (pT)**				**<0.001**
T1 ( < 2 cm)	77 (39.7%)	136 (61.5%)	213 (51.3%)	
T2 (2-5 cm)	102 (52.6%)	81 (36.7%)	183 (44.1%)	
T3 ( > 5 cm)	15 (7.7%)	4 (1.8%)	19 (4.6%)	
**Lymph node status (pN)**				**<0.001**
Positive	116 (59.8%)	82 (37.1%)	198 (47.7%)	
Negative	73 (37.6%)	132 (59.7%)	205 (49.4%)	
Missing	5 (2.6%)	7 (3.2%)	12 (2.9%)	
**Tumor grade**				**<0.001**
High	145 (74.7%)	105 (47.5%)	250 (60.2%)	
Low-intermediate	49 (25.3%)	113 (51.1%)	162 (39.0%)	
Missing	0 (0%)	3 (1.4%)	3 (0.7%)	
**Subtype**				0.86
ER + /HER2-	118 (60.8%)	146 (66.1%)	264 (63.6%)	
TNBC	50 (25.8%)	51 (23.1%)	101 (24.3%)	
HER2+	26 (13.4%)	24 (10.9%)	50 (12.0%)	

Cases were defined as patients who developed distant recurrence during study period and controls were patients who did not develop distant recurrence during study period.

Wilcoxon rank sum test is used for continuous variables. Chi-squared tests or Fisher's exact tests are used for categorical variables. Three patients were excluded as they were missing distant recurrence data. *SD* Standard deviation, *pT* pathological tumor stage, *pN* pathological lymph node status, *ER+* Estrogen receptor positive, *TNBC* Triple negative breast cancer.

**Table 2:** Patient characteristics, by subtype

	ER + /HER2-			TNBC			HER2 +		
	White	Black	*P*-value	White	Black	P-value	White	Black	*P*-value
	(*N* = 99)	(*N* = 101)		(*N* = 24)	(*N* = 60)		(*N* = 22)	(*N* = 23)	
**Institution**			**<0.001**			**0.07**			0.07
Cornell	16 (16.2%)	0 (0%)		0 (0%)	0 (0%)		2 (9.1%)	0 (0%)	
Montefiore	16 (16.2%)	48 (47.5%)		7 (29.2%)	34 (56.7%)		3 (13.6%)	8 (34.8%)	
MSKCC	52 (52.5%)	44 (43.6%)		16 (66.7%)	24 (40.0%)		9 (40.9%)	12 (52.2%)	
NYU	15 (15.2%)	9 (8.9%)		1 (4.2%)	2 (3.3%)		8 (36.4%)	3 (13.0%)	
**Distant recurrence**			**0.05**			0.92			**0.007**
**Yes**	53 (53.5%)	39 (38.6%)		13 (54.2%)	30 (50.0%)		16 (72.7%)	7 (30.4%)	
**No**	46 (46.5%)	62 (61.4%)		11 (45.8%)	30 (50.0%)		6 (27.3%)	16 (69.6%)	
**Age** (**years)**			0.64			0.86			0.56
Mean (SD)	56.2 (13.5)	57.3 (13.9)		60.0 (15.5)	59.0 (11.9)		56.4 (13.9)	54.1 (11.3)	
Median	56	58		63	57		55.5	55	
[Min, Max]	[26.0, 89.0]	[29.0, 88.0]		[36.0, 89.0]	[36.0, 84.0]		[30.0, 76.0]	[34.0, 73.0]	
**Body mass index**			**0.02**			**<0.001**			0.08
Mean (SD)	27.6 (6.42)	29.3 (5.98)		24.4 (4.10)	32.3 (6.69)		29.3 (6.34)	33.9 (8.10)	
Median	26.3	28.3		23.2	32.1		30.3	32.7	
[Min, Max]	[18.7, 51.7]	[15.4, 43.9]		[19.1, 32.8]	[21.1, 56.8]		[17.5, 41.0]	[22.0, 51.1]	
Missing	17 (17.2%)	0 (0%)		0 (0%)	1 (1.7%)		2 (9.1%)	2 (8.7%)	
**Time to distant recurrence (months)**			**0.02**			0.84			0.06
Mean (SD)	53.9 (35.3)	36.9 (33.0)		24.4 (21.5)	25.9 (20.2)		54.9 (36.6)	29.0 (24.5)	
Median	51	30		14	19.5		43	28	
[Min, Max]	[3, 163]	[0, 133]		[1, 77]	[2, 89]		[2, 136]	[0, 60]	
**Surgery type**			0.4			0.11			0.33
BCT/Lumpectomy	56 (56.6%)	64 (63.4%)		14 (58.3%)	43 (71.7%)		8 (36.4%)	13 (56.5%)	
Lumpectomy followed by mastectomy	1 (1.0%)	0 (0%)		1 (4.2%)	6 (10.0%)		1 (4.5%)	2 (8.7%)	
Mastectomy	41 (41.4%)	37 (36.6%)		9 (37.5%)	10 (16.7%)		13 (59.1%)	8 (34.8%)	
Missing	1 (1.0%)	0 (0%)		0 (0%)	1 (1.7%)		0 (0%)	0 (0%)	
**Radiation**			1			0.23			0.22
Yes	67 (67.7%)	69 (68.3%)		16 (66.7%)	49 (81.7%)		10 (45.5%)	16 (69.6%)	
No	27 (27.3%)	28 (27.7%)		8 (33.3%)	11 (18.3%)		11 (50.0%)	7 (30.4%)	
Missing	5 (5.1%)	4 (4.0%)		0 (0%)	0 (0%)		1 (4.5%)	0 (0%)	
**Tumor stage (pT)**			0.41			0.66			0.45
T1 ( < 2 cm)	48 (48.5%)	50 (49.5%)		14 (58.3%)	34 (56.7%)		8 (36.4%)	13 (56.5%)	
T2 (2–5 cm)	48 (48.5%)	44 (43.6%)		10 (41.7%)	24 (40.0%)		12 (54.5%)	9 (39.1%)	
T3 ( > 5 cm)	3 (3.0%)	7 (6.9%)		0 (0%)	2 (3.3%)		2 (9.1%)	1 (4.3%)	
**Lymph node status** (**pN)**			0.2			0.76			0.23
Positive	47 (47.5%)	58 (57.4%)		10 (41.7%)	29 (48.3%)		11 (50.0%)	8 (34.8%)	
Negative	49 (49.5%)	40 (39.6%)		14 (58.3%)	31 (51.7%)		9 (40.9%)	15 (65.2%)	
Missing	3 (3.0%)	3 (3.0%)		0 (0%)	0 (0%)		2 (9.1%)	0 (0%)	
**Tumor grade**			0.32			1			0.67
High	43 (43.4%)	53 (52.5%)		20 (83.3%)	54 (90.0%)		18 (81.8%)	20 (87.0%)	
Low-intermediate	54 (54.5%)	48 (47.5%)		3 (12.5%)	6 (10.0%)		4 (18.2%)	3 (13.0%)	
Missing	2 (2.0%)	0 (0%)		1 (4.2%)	0 (0%)		0 (0%)	0 (0%)	
**Follow up time (months)**			0.69			0.98			0.23
Mean (SD)	102 (46.4)	100 (50.6)		97.6 (56.7)	96.3 (59.2)		95.2 (42.7)	114 (47.7)	
Median	97.7	103		112	106		85.2	122	
[Min, Max]	[12, 198]	[3, 204]		[17, 165]	[10, 199]		[33, 182]	[30, 173]	

Wilcoxon rank sum test is used for continuous variables Chi-squared tests or Fisher’s exact tests are used for categorical variables Patients with missing data on distant recurrences were not included in analysis (1 patient)*SD*Standard deviation,*pT*pathological tumor stage,*pN*pathological lymph node status,*ER+*Estrogen receptor positive,*TNBC*Triple negetive breast cancer

**Table 3:** Patient characteristics, all subtypes

	White	Black	All Patients	*P*-value
	(*N* = 145)	(*N* = 184)	(*N* = 329)	
**Institution**				
**Cornell**	18 (12.4%)	0 (0%)	18 (5.5%)	
Montefiore	26 (17.9%)	90 (48.9%)	116 (35.3%)	
MSKCC	77 (53.1%)	80 (43.5%)	157 (47.7%)	
NYU	24 (16.6%)	14 (7.6%)	38 (11.6%)	
**Distant Recurrence**				0.008
Yes	82 (56.6%)	76 (41.3%)	158 (48.0%)	
No	63 (43.4%)	108 (58.7%)	171 (52.0%)	
**Age (years)**				0.77
Mean (SD)	56.9 (13.9)	57.5 (13.0)	57.2 (13.4)	
Median [Min, Max]	57.0 [26.0, 89.0]	57.0 [29.0, 88.0]	57.0 [26.0, 89.0]	
**Body Mass Index**				**<0.001**
Mean (SD)	27.3 (6.18)	30.8 (6.69)	29.4 (6.71)	
Median [Min, Max]	26.2 [17.5, 51.7]	30.2 [15.4, 56.8]	28.6 [15.4, 56.8]	
Missing	19 (13.1%)	3 (1.6%)	22 (6.7%)	
**Time to distant recurrence (months)**				0.06
Mean (SD)	49.4 (35.1)	31.8 (28.0)	40.9 (33.0)	
Median [Min, Max]	43.0 [1,163]	27.0 [0-133]	33.0 [0-163]	
**Surgery type**				0.03
BCT/Lumpectomy	78 (53.8%)	120 (65.2%)	198 (60.2%)	
Lumpectomy followed by mastectomy	3 (2.1%)	8 (4.3%)	11 (3.3%)	
Mastectomy	63 (43.4%)	55 (29.9%)	118 (35.9%)	
Missing	1 (0.7%)	1 (0.5%)	2 (0.6%)	
**Radiation**				0.18
Yes	93 (64.1%)	134 (72.8%)	227 (69.0%)	
No	46 (31.7%)	46 (25.0%)	92 (28.0%)	
Missing	6 (4.1%)	4 (2.2%)	10 (3.0%)	
**Tumor stage (pT)**				0.41
T1 ( < 2 cm)	70 (48.3%)	97 (52.7%)	167 (50.8%)	
T2 (2-5 cm)	70 (48.3%)	77 (41.8%)	147 (44.7%)	
T3 ( > 5 cm)	5 (3.4%)	10 (5.4%)	15 (4.6%)	
**Lymph node status (pN)**				0.56
Positive	68 (46.9%)	95 (51.6%)	163 (49.5%)	
Negative	72 (49.7%)	86 (46.7%)	158 (48.0%)	
Missing	5 (3.4%)	3 (1.6%)	8 (2.4%)	
**Tumor grade**				0.03
High	81 (55.9%)	127 (69.0%)	208 (63.2%)	
Low-intermediate	61 (42.1%)	57 (31.0%)	118 (35.9%)	
Missing	3 (2.1%)	0 (0%)	3 (0.9%)	
**Subtype**				0.003
ER + /HER2-	99 (68.3%)	101 (54.9%)	200 (60.8%)	
TNBC	24 (16.6%)	60 (32.6%)	84 (25.5%)	
HER2 +	22 (15.1%)	23 (12.5%)	45 (13.7%)	
**Follow up time (months)**				0.95
Mean (SD)	101 (47.4)	101 (53.2)	100 (50.7)	
Median [Min, Max]	96.8 [12, 198]	110 [3, 204]	103 [3, 204]	

Wilcoxon rank sum test is used for continuous variables Chi-squared tests or Fisher’s exact tests are used for categorical variables Patients with missing data on distant recurrences were not included in analysis (1 patient)*SD*Standard deviation,*pT*pathological tumor stage,*pN* pathological lymph node status,*ER+*Estrogen receptor positive,*TNBC* Triple negetive breast cancer

When stratified by cases versus controls, most baseline characteristics did not differ significantly between the groups; however, cases were associated with larger tumors, positive lymph nodes, and higher tumor grades (Table 1). Of note, one of the 265 White ER + /HER2− patients was missing distant recurrence data and, as such, was eliminated from the study. Upon stratification by breast cancer subtype and race, most clinicopathologic features, treatment regimens and follow-up times were similar between Black and White patients (Table 2). Within the ER + /HER2- subtype, a marginally lower proportion of Black patients developed distant recurrence than White patients (39% vs 54%, *p* = 0.05), but Black patients had earlier recurrences when compared to White patients (median 30 months vs 51 months, *p* = 0.02). In the HER2+ subtype, a lower proportion of Black patients had distant recurrences compared to White patients (30.4% vs 72.7%, *p* = 0.007). In the TNBC subtype, no significant differences in distant recurrences were observed between races. Black patients compared to White exhibited higher BMI in ER + /HER2- (mean 29.3 vs 27.6 kg/m^2^, *p* = 0.02) and TNBC (mean 32.3 vs 24.4 kg/m^2^, *p* < 0.001) subtypes, but no BMI differences were seen in the HER2+ subtype (Table 2).

### Disparities in pro-metastatic tumor microenvironment parameters exist across breast cancer subtypes in treatment-naïve breast cancer

**Fig. 2:**
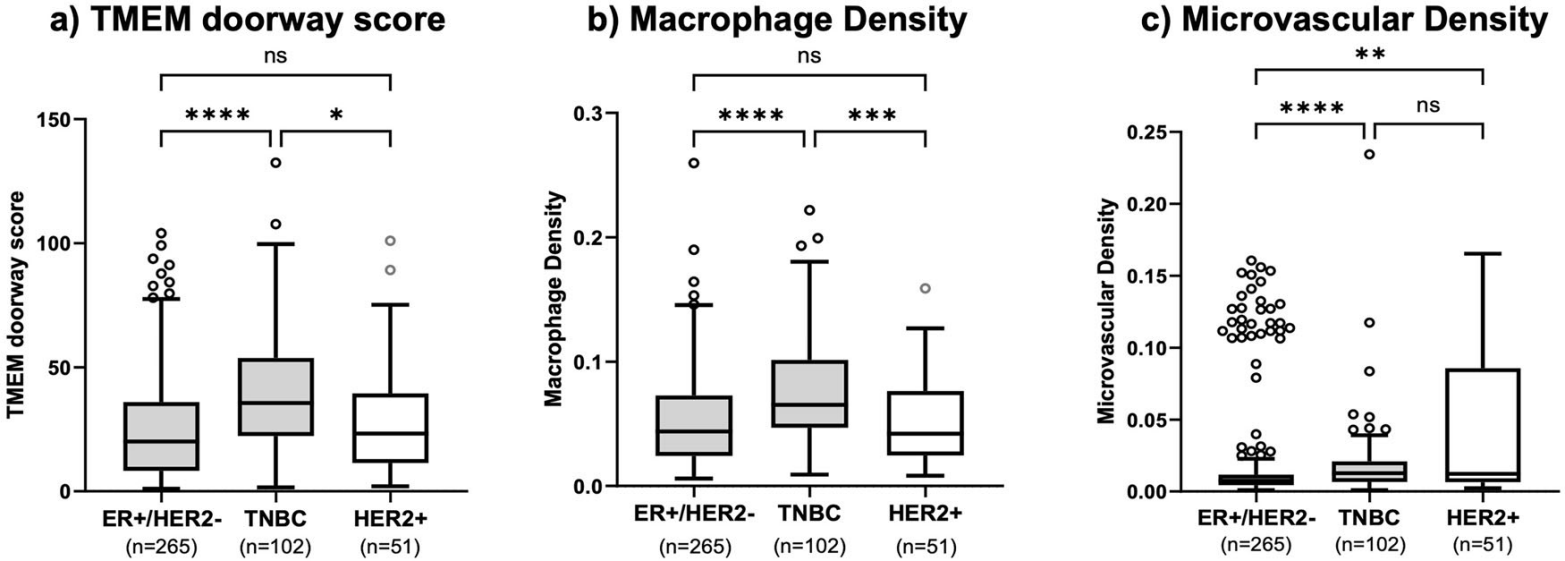
Tumor Microenvironment (TME) parameters by breast cancer subtype (all cases and controls). **a** TMEM Doorway score and **b** macrophage density were all higher in patients with TNBC (*n* = 102), compared to ER + /HER2- breast cancer (*n* = 265) and HER2+ (*n* = 51). **c** microvascular density was significantly higher in TNBC and HER2+ compared to ER + /HER2- breast cancer. Outliers are single data points more than 1.5 times of upper and lower quartiles.

In the overall study population, TNBC tumors had significantly higher TMEM doorway scores compared to ER + /HER2- (median 35.6 vs 20.1, *p* < 0.001) and HER2+ tumors (median 35.6 vs 23.3, *p* = 0.03) (Fig. 2a). TNBC also showed higher macrophage densities than ER + /HER2- (median 0.07 vs 0.04, *p* < 0.001) and HER2+ tumors (median 0.07 vs 0.04, *p* < 0.001) (Fig. 2b). Microvascular density was significantly higher in both TNBC and HER2+ tumors compared to ER + /HER2- (TNBC: median 0.012 vs 0.007, *p* < 0.001; HER2 + : median 0.012 vs 0.007, *p* = 0.002) (Fig. 2c). Similar results were observed when comparisons were confined to controls (patients who did not develop metastases, Supplementary Fig. 1).

**Fig. 3:**
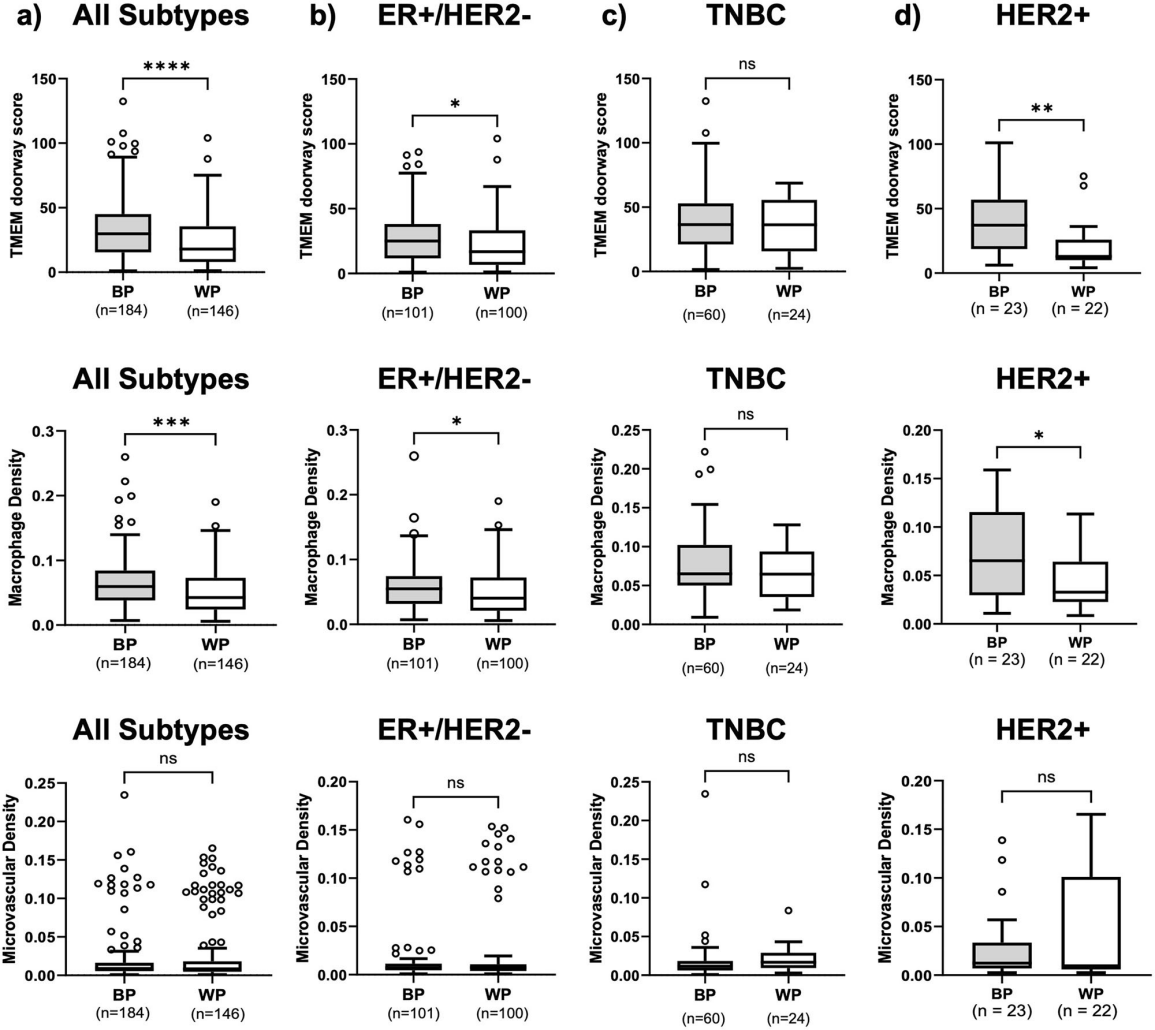
Racial disparities in TMEM doorway score and macrophage density in treatment-naïve tissues. Racial differences in TMEM doorway score and macrophage density were observed in **a** the entire cohort, **b** the ER + /HER2- group, and **d** HER2+ group, but not in **c** the TNBC group. There was no racial differences in macrophage density in any of the groups. Outliers represent data points more than 1.5 times above or below the upper and lower quartiles.

We next examined whether racial differences in TME parameters exist across breast cancer subtypes. Combining all subtypes, Black compared to White patients had significantly higher TMEM doorway scores (median 29.9 vs 17.9, *p* < 0.001) and higher macrophage densities (median 0.06 vs 0.04, *p* < 0.001) (Fig. 3a). When stratified by subtype, Black, compared to White patients, with ER + /HER2- tumors had higher TMEM doorway scores (median 25.0 vs 16.8, *p* = 0.01) and macrophage densities (median 0.05 vs 0.04, *p* = 0.02) (Fig. 3b)**;** Similarly, in HER2+ tumors, Black compared to White patients had higher TMEM doorway scores (median 37.2 vs 12.9, *p* = 0.003) and macrophages densities (median 0.07 vs 0.03, *p* = 0.04) (Fig. 3d). No significant racial differences were observed in TNBC (Fig. 3c). Microvascular density did not differ by race in any of the subtypes (Fig. 3a–d).

Together, these results indicate that while TNBC tumors are enriched for pro-metastatic TME parameters – particularly TMEM doorways and macrophage density – racial differences in these markers are primarily observed in the ER + /HER2- and HER2+ subtypes, with Black patients having higher levels compared to White patients.

### Racial differences in pro-metastatic TME parameters are associated with odds of distant recurrence

To evaluate the clinical impact of racial differences on distant recurrence, we performed an univariable logistic regression analysis assessing the association between clinical, pathological variables and distant recurrence during the entire follow-up period. Given our prior observation that TMEM doorway score was found to have a time dependent association for distant relapse (significant within the first 5 years, but not for longer periods)^21^, we also performed our analysis within 5-year and 10-year follow up periods. Logistic regression models were fitted separately for each predictor variable (Table 4). Among ER + /HER2- patients evaluated over the entire study follow up period (range 3 – 204 months), 69% of Black patients (*n* = 70/101) and 62% of White patients (*n* = 55/100) had high TMEM doorway scores (≥15, see methods). Tumor stage ≥20 mm vs <20 mm (OR 3.30, 95% CI 1.91-5.71, *p* < 0.001), positive vs negative lymph nodes (OR 3.67, 95% CI 2.08-6.47, *p* < 0.001), and high vs low tumor grade (OR 4.86, 95% CI 3.76–8.56, *p* < 0.001) were all significantly associated with higher odds of distant recurrence. However, Black vs White race (*p* = 0.41), high vs low TMEM doorway scores (*p* = 0.38) and high vs low macrophage density scores (*p* = 0.90) were not significantly associated with increased odds of distance recurrence. When analyzed for 5- and 10-year follow up periods, no association with distance recurrence was seen for Black vs White race (*p* = 0.49, *p* = 0.44), high vs low TMEM doorway score (*p* = 0.44, *p* = 0.44), or high vs low macrophage density (*p* = 0.84, *p* = 0.88) (Table 4). Due to the small sample size, this analysis was not performed for the TNBC and HER2+ subtypes.

**Table 4:** Univariable analysis of risk factors for distant recurrence in ER + /HER2- breast cancer across all years, 0-5 years and 0-10 years

	All Years		0 – 5 Years		0 – 10 Years	
Variables	OR (95% CI)	*p*-value	OR (95% CI)	*p*-value	OR (95% CI)	*p*-value
Age (years)	0.99 (0.97 – 1)	0.13	0.99 (0.97 - 1.01)	0.53	0.99 (0.97 - 1.01)	0.32
Body Mass Index ≥ 30 (ref: <30)	0.96 (0.55 - 1.66)	0.88	0.94 (0.52 - 1.7)	0.84	1.07 (0.62 - 1.86)	0.8
Black race (ref: white)	0.78 (0.43 - 1.41)	0.41	1.25 (0.67 - 2.34)	0.49	0.79 (0.44 - 1.44)	0.44
Surgery Type: mastectomy (ref: BCT/lumpectomy)	1.55 (0.9 - 2.64)	0.11	1.47 (0.83 - 2.60)	0.18	1.63 (0.95 - 2.79)	0.08
Radiation (ref: no radiation)	0.61 (0.34 - 1.1)	0.1	0.66 (0.36 - 1.23)	0.19	0.60 (0.33 - 1.08)	0.09
**Tumor Stage (≥ 20** **mm) (ref:** < 20 **mm)**	**3.30** (**1.91 - 5.71)**	**<0.001**	**3.22** (**1.78 - 5.83)**	**<0.001**	**3.64** (**2.08 - 6.37)**	**<0.001**
**Positive lymph node (ref: negative)**	**3.67** (**2.08 - 6.47)**	**<0.001**	**2.60** (**1.43 - 4.73)**	**0.002**	**3.19** (**1.81 - 5.62)**	**<0.001**
**High tumor Grade (ref: low-intermediate)**	**4.86** (**2.76 - 8.56)**	**<0.001**	**4.26** (**2.33 - 7.79)**	**<0.001**	**4.93** (**2.78 - 8.73)**	**<0.001**
High TMEM Doorway Score (ref: low)	1.28 (0.73 - 2.24)	0.38	1.27 (0.7 - 2.32)	0.44	1.25 (0.71 - 2.20)	0.44
High Macrophage Density (ref: low)	1.04 (0.58 - 1.87)	0.9	0.94 (0.5 - 1.77)	0.84	0.96 (0.53 - 1.74)	0.88

Each variable’s impact on risk of distant recurrence was analyzed individually using logistic regression models.

**Fig. 4:**
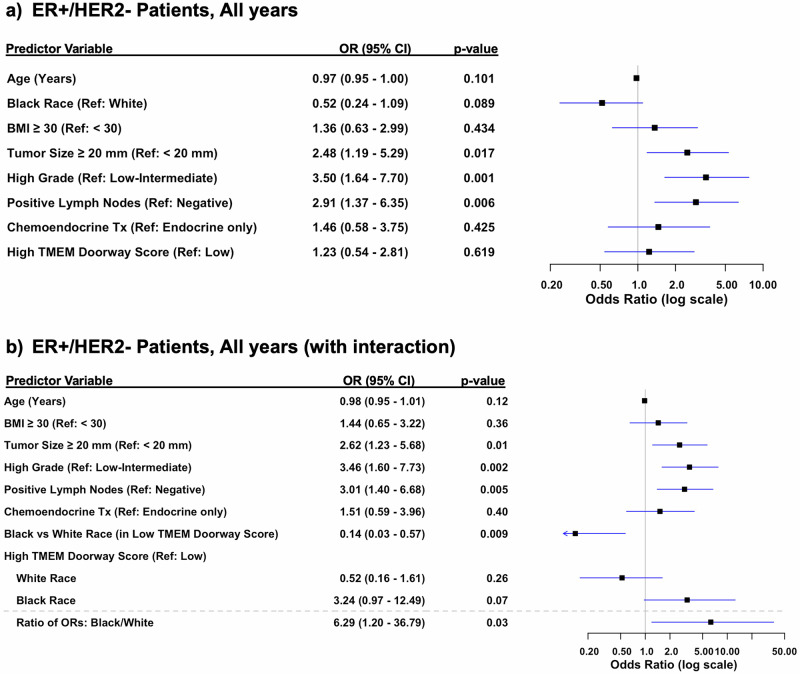
Multivariable analysis of risk factors for distant recurrence, adjusting for clinicopathologic variables. **a** Forest Plot of multivariable logistic regression modeling for odds of distant recurrence in ER + /HER2- over all years, adjusting for clinicopathological variables. **b** Forest Plot of multivariable logistic regression modeling for odds of distant recurrence in ER + /HER2-, all years, adjusting for clinicopathological variables and including terms for treatment and the interaction between race and high TMEM doorway score.

**Fig. 5:**
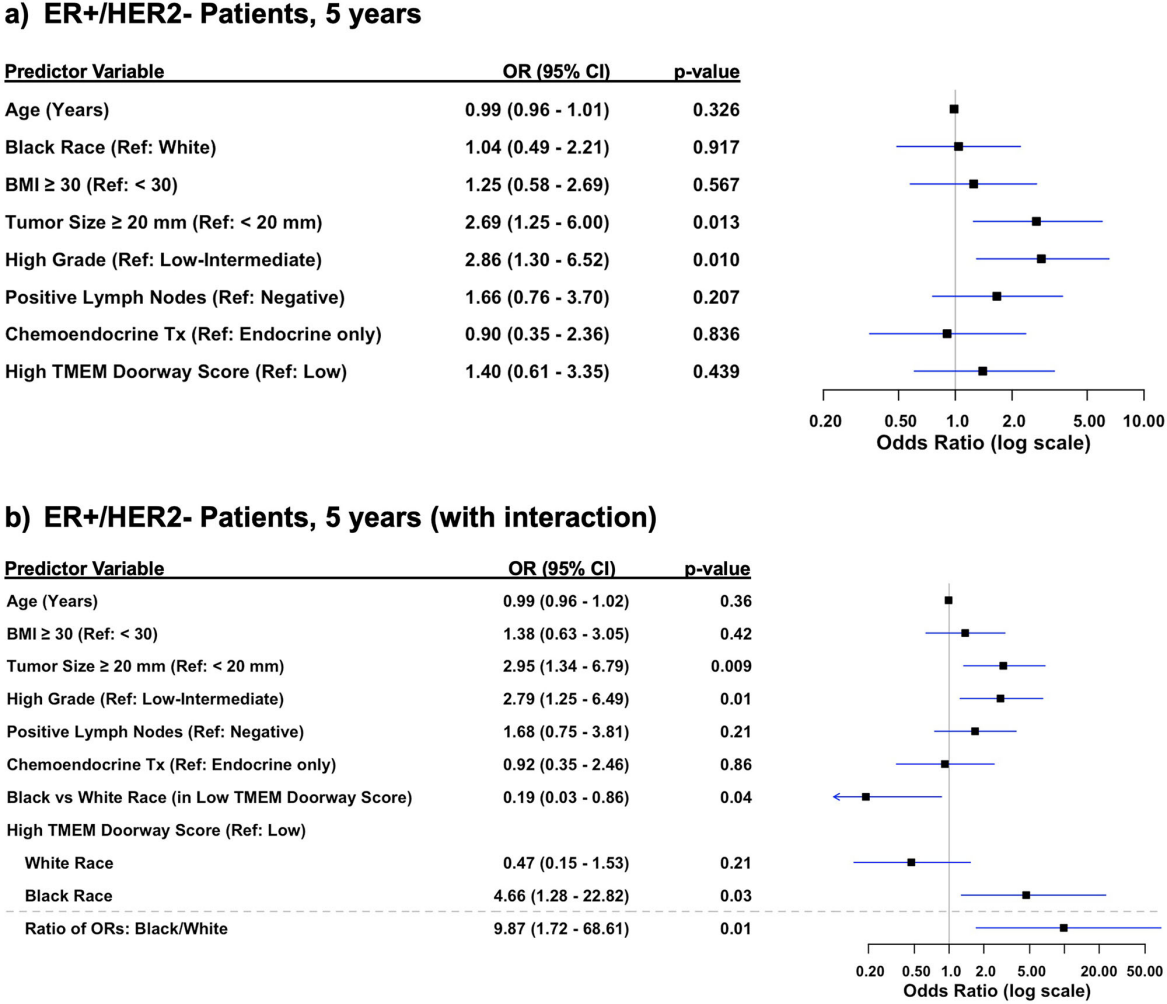
Multivariable analysis of risk factors for distant recurrence, adjusting for clinicopathologic variables in the first five years. **a** Forest Plot of multivariable logistic regression modeling odds of distant recurrence in ER + /HER2- over first 5 years, adjusting for clinicopathological variables. **b** Forest Plot of multivariable logistic regression with interaction between race and high TMEM doorway score. Black patients with high TMEM Doorway scores had significantly higher odds of distant recurrence after adjustment for other clinicopathologic variables. White patients with high TMEM doorway scores were less likely to experience higher odds of distant recurrence compared to white patients with low TMEM doorway scores after adjusting for age, BMI, tumor size, lymph node status, grade, and treatment.

**Fig. 6:**
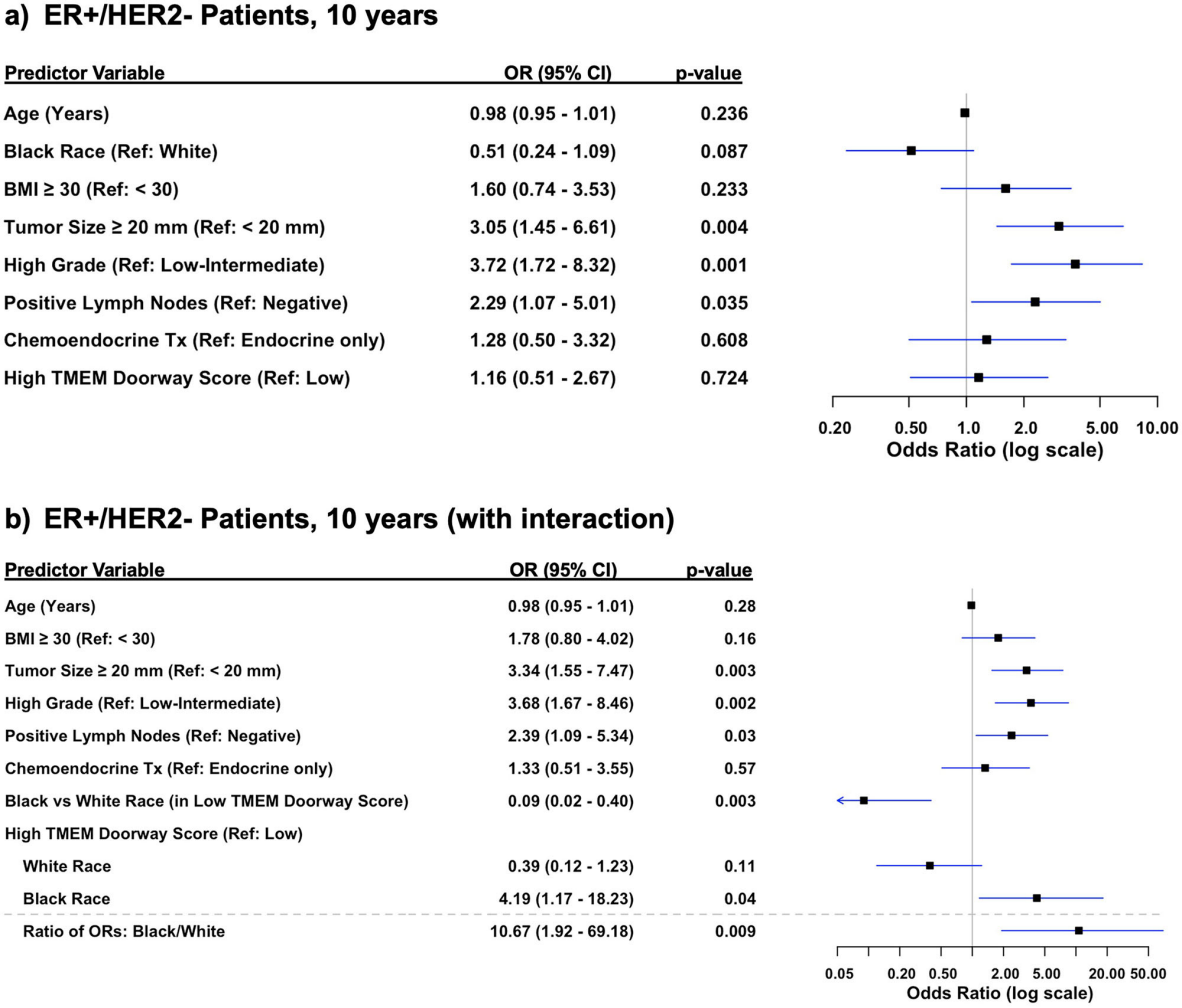
Multivariable analysis of risk factors for distant recurrence, adjusting for clinicopathologic variables in the first ten years. **a** Forest Plot of multivariable logistic regression modeling odds of distant recurrence in ER + /HER2- over first 10 years, adjusting for clinicopathological variables. **b** Forest Plot of multivariable logistic regression with interaction between race and high TMEM doorway score. Black patients with high TMEM Doorway scores had significantly higher odds of distant recurrence after adjustment for other clinicopathologic variables. White patients with high TMEM doorway scores were less likely to experience higher odds of distant recurrence compared to white patients with low TMEM doorway scores after adjusting for age, BMI, tumor size, lymph node status, grade, and treatment.

To control for confounders, we performed a multivariable logistic regression analysis, adjusting for patient’s age, race, BMI, tumor size, lymph node status, grade, and treatment (Figs. 4–6**)**. Because this was a retrospective study and data influencing clinical decision-making (such as Oncotype scores) were not consistently available for all patients, treatment for ER + /HER2- patients was included as a covariate. This inclusion accounts for variability in treatment decisions wherein patients considered high-risk typically receive chemoendocrine therapy, while those with lower clinical risk receive endocrine therapy alone. Our multivariable model for ER + /HER2- patients showed that tumor size ≥20 mm vs <20 mm (OR 2.48, 95% CI 1.19–5.29; *p* = 0.02), positive lymph nodes vs negative lymph nodes (OR 2.9, 95% CI 1.37–6.35; *p* = 0.006), and high grade tumors vs low grade (OR 3.5, 95% CI 1.64–7.70; *p* = 0.001) were all statistically significant predictors of distant recurrence (Fig. 4a). Additionally, when we limited follow up to the first 5- or first 10-year follow up periods, tumor size ≥20 mm vs <20 mm (OR 3.05 95% CI 1.45–6.61; *p* = 0.004), high grade vs low grade tumors (OR 3.72, 95% CI 1.72–8.32; *p* = 0.001), and positive vs negative lymph nodes (OR 2.29, 95% CI 1.07 - 5.01; *p* = 0.035) remained significant at 10-years, whereas only tumor size ≥20 mm vs <20 mm (OR 2.69 95% CI 1.25–6.00; *p* = 0.013) and high grade vs low grade tumors (OR 2.86, 95% CI 1.30–6.52; *p* = 0.010) remained significant at 5-years (Figs. 5a & 6a).

Given the observed racial differences in TMEM doorway scores — specifically, that Black patients had higher scores — we included an interaction term between race and TMEM doorway scores in our analysis for ER + /HER2- patients (Figs. 4b, 5b and 6b). This aimed to evaluate whether the prognostic impact of high TMEM doorway scores on distant recurrence outcomes varied by race over the entire study period (Fig. 4b) and at 5-(Fig. 5b) and 10-year intervals (Fig. 6b). Over the entire study period, Black patients with high TMEM doorway score had higher odds of distant recurrence compared to those with low TMEM doorway scores (OR 3.24, 95% CI 0.97–12.49, *p* = 0.07), with borderline significance (Fig. 4b). When limiting the follow-up to the first 5 years we showed that Black patients with high TMEM doorway scores were consistently more likely to experience a distant recurrence, with statistical significance (OR 4.6, 95% CI 1.28–22.82, *p* = 0.03), compared to Black patients with low TMEM doorway scores (Fig. 5b). In contrast, no significant association was observed for White patients with high TMEM doorway scores compared to White patients with low TMEM doorway scores (*p* = 0.21) after adjusting for age, BMI, tumor size, lymph node status, grade, and treatment (Fig. 5b). At 10 years, a similar result was observed; Black patients with high TMEM doorways scores were more likely to have a distant recurrence (OR 4.2, 95% CI 1.17–18.23, *p* = 0.04) compared to those with low TMEM doorway scores and this difference was not seen among the White patients (*p* = 0.11) (Fig. 6b). Importantly, across each follow up period, race and high TMEM doorway score had a statistically significant interaction (Figs. 4b, 5b and 6b): all years of follow up (ratio of ORs: Black/White with high TMEM doorway score 6.29, 95% CI 1.20–36.79, *p* = 0.03), at 5 years (ratio of ORs: Black/White with high TMEM Doorway score 9.87, 95% CI 1.72– 68.61, *p* = 0.01), and at 10 years (ratio of ORs: Black/White with high TMEM Doorway score 10.67, 95% CI 1.92–69.18, *p* = 0.009). Additionally, in this model, Black (compared to White) patients with low TMEM doorway scores had significantly lower odds of distant recurrence through the entire study follow up period (OR 0.14, 95% CI 0.03–0.57, *p* = 0.009), at 5 years (OR 0.19, 95% CI 0.03–0.86, *p* = 0.04), and at 10 years (OR 0.09, 95% CI 0.02– 0.40, *p* = 0.003) (Figs. 4b, 5b, 6b).

To assess whether TMEM doorway score represents a distinct prognostic marker beyond overall macrophage density, we conducted a multivariable logistic regression analysis using macrophage tertiles (high vs. low-intermediate), adjusting for age, BMI, tumor size, grade, nodal status, and treatment (Supplementary Table 1). In Model 1, high macrophage density was not independently associated with increased odds of distant recurrence (OR 0.70, 95% CI 0.30–1.58, *p* = 0.40). In Model 2, we tested for an interaction between Black race and high macrophage density. The interaction term was not statistically significant (OR 3.61, 95% CI 0.68–20.87, *p* = 0.139), indicating that Black patients with high macrophage density did not have significantly higher odds of recurrence compared to White patients with similarly high macrophage levels, after adjusting for clinicopathologic variables.

## Discussion

In this retrospective, multi-institutional study, we examined racial differences in pro-metastatic tumor microenvironment (TME) parameters across breast cancer subtypes in treatment-naïve patients. Furthermore, we assessed the association between TMEM doorway score and risk of distant recurrence in patients with ER + /HER2- breast cancer.

Our results revealed that TNBC tumors exhibited a more pro-metastatic microenvironment, with significantly higher TMEM doorway score and macrophage density compared to ER + /HER2- and HER2+ breast cancers. Strikingly, Black patients with ER + /HER2- and HER2+ breast cancer had significantly higher TMEM doorway score and macrophage density than White ER + /HER2- and HER2+ patients, a difference not observed in TNBC. Additionally, in ER + /HER2- breast cancer, there was a significant difference between TMEM doorway score and risk of distant recurrence when analysis was adjusted by race. Black patients with high TMEM doorway scores had a significantly higher risk of distant recurrence compared to White patients with similar scores.

Taken together, these findings suggest that the TNBC subtype has an intrinsically pro-metastatic TME without any racial differences, but for ER + /HER2- breast cancer, Black patients, compared to White patients, face a significantly more unfavorable TME, which contributes to increased risk of distant recurrence. This underscores the critical interplay of race, biology, and clinical outcomes in breast cancer.

Our findings align with existing literature that highlights Black race as an important factor in breast cancer disparities, particularly for the ER + /HER2- subtype^8^. Previously, we showed that, post neoadjuvant chemotherapy, Black patients with residual ER + /HER2- disease have higher TMEM doorway scores and macrophage density compared to White patients^9^. Here, we extend those findings to treatment-naïve tumors, demonstrating that the previously discovered disparities in pro-metastatic TME exist at baseline. These findings suggest that biological differences may contribute to racial disparities even before the treatment is initiated. Notably, higher TMEM doorway density may be an underexplored biological driver of early metastatic recurrence and poor survival, even in patients with similar OncotypeDX scores, as suspected previously^21^. Our new findings add to the growing body of evidence that racial disparities in breast cancer outcomes (especially in the ER + /HER2- subtype) may have a biological basis, in part due to differences in the TME.

### Implications of racial disparities in ER + /HER2- breast cancer

Racial disparities in breast cancer outcomes are often attributed to socioeconomic factors and access to care^1–5^. Our study, however, highlights the important role biological differences in the TME play in racial disparities in outcomes. This aligns with previous large-scale clinical trials (involving close to 20,000 patients) showing worse survival for African American patients with sex-specific cancers (such as ER+ breast cancer), despite uniform stage, treatment, and follow-up^2^.

Here, we observe significant interaction between race and TMEM doorway scores in ER + /HER2- patients. Black patients with high scores had higher odds of distant recurrence compared to White counterparts, suggesting a baseline biological disadvantage. One possible explanation is that TMEM doorway score may simply reflect more aggressive tumor features such as high grade or large tumor size, which are more common in Black patients. While we adjusted for these clinicopathologic variables in multivariable models, we cannot fully exclude the possibility that TMEM doorway score serves as a surrogate for unmeasured features of aggressive disease. However, our prior studies have shown that TMEM doorway score is prognostic for distant recurrence specifically in the first 5 years of ER + /HER2- breast cancer in multiple clinical cohorts^20,21^, as well as it being independent of genomic assay, oncotype score, indicating a independent prognostic indicator of metastasis despite intrinsic tumor biology features. The persistence of the race × TMEM doorway score interaction after adjustment, and its specificity to ER + /HER2− tumors, is consistent with our previous studies.

Additionally, our findings expand on the Sparano et al. study^21^, which examined the prognostic role of TMEM doorway score in 600 patient samples taken from the ECOG E2197 trial. This study found that, for ER + /HER2- patients (297 of the 600), higher TMEM doorway score is prognostic for distant recurrence risk in the first 5 years, but not for the first 10 years^21^.

Notably, we did not observe a statistically significant association between high TMEM doorway score and distant recurrence when both races were included (Fig. 4a). This is likely due to our smaller overall sample size, underpowering our study to detect this difference in the entire population. Although the association between TMEM doorway score and recurrence was not significant in the overall cohort, we acknowledge this may not be due to limited power alone. TMEM appeared prognostic at 5 years but not at later time points, which may suggest that its impact diminishes over time. Additionally, we saw a strong association in Black patients but not in White patients, raising the possibility that pooling groups could obscure race specific effects.

The demographic makeup of the Sparano study, which consisted predominantly of White patients (>90%), precluded an evaluation of racial differences. By contrast, our balanced racial distribution (49% White, 51% Black) allowed us to analyze race’s influence on TMEM doorway scores and distant recurrence.

Thus, though we found no statistically significant difference between high TMEM doorway score and distant recurrence when considering all races combined, our interaction model revealed that race modifies the relationship between TMEM doorway and metastatic risk, despite this smaller sample size. This suggests that high TMEM doorway scores may have a more severe impact (larger effect size) on metastatic outcomes in Black patients compared to White patients. It is important to note the wide confidence intervals in the interaction model, likely due to small sample sizes. While the findings remain significant, caution is needed in interpreting the magnitude of the effect.

Finally, in our model, we observed that Black (compared to White) ER + /HER2- patients with low TMEM doorway scores had a reduced odd of distant recurrence, indicating that their baseline biology may be protective against metastatic dissemination. This result poses the possibility that insights from this patient population (particularly in comparison to TMEM doorway high Black/White patients, or to TMEM doorway low White patients) could help understand what baseline biological factors result in reduced dissemination risk. Future studies investigating the unique tumor microenvironment of this population of patients are warranted.

Our results thus underscore the need for a deeper exploration of how biological factors (such as the TME) not only differ between races, but also lead to disparities in breast cancer prognosis. Therefore, our findings highlight the importance of addressing racial disparities in breast cancer by targeting the underlying biology in addition to healthcare inequities.

### The role of the tumor microenvironment in racial disparities

The TME (comprising various cellular components like fibroblasts, adipocytes, immune cells, and endothelial cells, along with a myriad of signaling molecules and extracellular matrix (ECM) elements) shapes breast cancer progression and influences outcomes. In Black patients, the TME is often characterized by a higher density of pro-tumorigenic immune cells, such as M2 macrophages and regulatory T cells, along with increased microvascular density^13^. These features, coupled with the intricate interactions between cancer cells and stromal elements, promote invasive tumor growth and contribute to disparities in outcomes between Black and White patients^13,22,23^.

At the genomic level, racial differences in the TME have also been noted. Several studies have shown that populations with geographic ancestries exposed to environmental pathogens often have variants in innate immunity genes^13,24^. While these variants provide protection from infection, they may also predispose individuals to worse cancer outcomes^13^. In one study, tumors from American women of African ancestry displayed higher microvascular density and macrophage infiltration compared to tumors from American women of European ancestry, indicating differences in baseline enhanced tumor angiogenesis and chemotaxis pathways^25^. Our findings of higher TMEM doorway score and macrophage density in Black patients with ER + /HER2- breast cancer compared to White patients contribute to this growing understanding of how the TME-driven racial disparities impact clinical outcomes.

### Clinical implications and future directions

Our findings underscore the potential utility of TMEM doorway scoring in the early risk stratification of Black patients with ER + /HER2- breast cancer. By identifying patients with elevated TMEM doorway densities, clinicians could more effectively tailor treatment strategies to mitigate the elevated recurrence risk in these patients. Additionally, *TMEM Activity-MRI (TDAM)*, a novel clinical imaging technique that detects TMEM-associated vascular openings, could enable real-time monitoring of TMEM activity, potentially guiding therapeutic decisions^26,27^.

Targeted therapies, such as the TIE2 inhibitor Rebastinib, which specifically targets TMEM doorway activity, hold promise for reducing distant recurrence in patients with high TMEM doorway scores. A recent phase 1b clinical trial showed no dose limiting toxicities for Rebastinib, while patients who received Rebastinib in combination with systemic therapies showed a significant decline in circulating tumor cells^28^. When coupled with early TMEM doorway detection by TDAM, such interventions may help address the biological drivers of racial disparities in breast cancer outcomes.

### Strength and limitations

This study’s strengths, including its large overall size (418 patients), multi-institutional sample collection, and inclusion of patients of different racial backgrounds. Although the power of the regression analysis was limited by a smaller subset of 200 ER + /HER2- patients, these factors collectively enabled a robust analysis of racial disparities in the TME. Additionally, our use of multivariate logistic regression allowed us to adjust for potential confounders, providing clearer insights into the relationship between race, TMEM doorways, and metastatic outcome. However, we do note some limitations. Treatment status was unknown for some patients, and comorbidities other than BMI (such as hypertension) were not accounted for, which could influence pro-metastatic markers and survival outcomes. Furthermore, the impact of social determinants of health on access to timely and appropriate treatment was not fully captured in this study. However, the study by Albain et al^2^. showed that in hormone dependent cancers such as ER+ breast cancer there is disparity in outcome even when the social determinants of health are taken into consideration. Additionally, while this study focused on macrophage density and TMEM doorway formation, we acknowledge that the tumor microenvironment includes other immune cell types such as cytotoxic and regulatory T cells, natural killer (NK) cells, and neutrophils, which may contribute to racial disparities but were not evaluated due to tissue and technical constraints. We used CD68 to capture total macrophage density, which does not distinguish between M1 and M2 phenotypes. Given the limitations of defining macrophage polarization, we prioritized the functional role of macrophages within TMEM structures. Future studies using are needed to better characterize immune phenotypes. Additionally, granular treatment data (including chemotherapy regimen, endocrine therapy duration, and adherence), which were not consistently available and hence not included, should be incorporated in future research. Additionally, we chose not to match on clinical variables like tumor size, nodal status, or treatment, as doing so would have limited our ability to evaluate their independent associations with recurrence and reduced the generalizability of our findings. Instead, we addressed potential confounding by adjusting for these key prognostic factors in our multivariable logistic regression model. We also reviewed the baseline distribution of clinical characteristics across recurrence status and found no major imbalances that would suggest residual confounding (see Table 2). The only significant difference was BMI, which was accounted for in the model. Our main findings, including the significant interaction between race and TMEM doorway score, remained robust after adjustment. For these reasons, we did not conduct additional matching or stratified analyses, though future prospective matched studies could be valuable to confirm these findings. While we adjusted for tumor size, grade, nodal status, and treatment, TMEM score may still reflect unmeasured aggressive biology more prevalent in some populations. Due to limited recurrence events in the TNBC and HER2+ subtypes, we were underpowered to perform subtype specific biomarker analyses among patients who recurred. Future studies with larger recurrence numbers are needed to evaluate whether TMEM doorway, macrophage, and microvascular density provide subtype specific prognostic value. Finally, self-reported race is a social construct and dividing women into Black and White race can not only underestimate the impact of social factors, but also may not accurately reflect the extent of genetic mix within an individual [29]. Therefore, studies that stratify patients by genetic ancestry may help identify additional factors contributing to these racial disparities in the TME.

In conclusion, this study identifies high TMEM doorways as a critical biological factor contributing to racial disparities in breast cancer outcomes, particularly among Black patients with ER + /HER2- breast cancer. Black patients with high TMEM doorway scores were more likely to develop distant metastases, suggesting that TMEM doorway score may underlie a biological basis for worse outcomes in Black patients with ER + /HER2- breast cancer. Interventions targeting TMEM doorways may potentially reduce these disparities and improve survival outcomes for Black women with breast cancer. Future studies should focus on integrating TMEM doorway score into clinical risk assessments and exploring TMEM doorway targeted therapies to improve outcomes for Black women with breast cancer.

## Methods

### Study design

This study was a multi-institutional unmatched case-control study performed in accordance with REMARK guidelines^29,30^. Patient samples were collected from New York Pathology Oncology Group institutions (NYPOG - https://einsteinmed.edu/research/groups/ny-pathology-oncology/) (Montefiore Einstein Comprehensive Cancer Center; Memorial Sloan Kettering Cancer Center; New York-Presbyterian/Weill Cornell Medical Center; and NYU Langone Health). The study was approved by the Institutional Review Board (IRB) of each institution. Written consent was not collected during this study, as the study exclusively used leftover archival formalin fixed paraffin embedded (FFPE) blocks excised years prior for standard clinical purposes.

Self-identified race was used, according to patients’ medical records. Patients in the “other race” group included those reported as Asian (*N* = 11) or other (*N* = 77) which including multi-racial or unknown race. Due to the low number patients in Asian race, and heterogeneity in other race, these patients were included in the other race group but were not included in multivariable analysis.

### Selection of cases and controls

The intent of this study was to include approximately equal numbers of cases with and without distant recurrence to ensure meaningful statistical comparison. Cases were defined as patients with localized breast cancer who subsequently developed distant recurrence, whereas controls were defined as patients who did not develop distant recurrence. Distant recurrence was defined as clinical or radiographic evidence of disease recurrence outside of the breast, chest wall, and axillary lymph nodes, with or without tissue confirmation.

The tumor registry at each institution was queried to identify eligible cases and controls based on pre-defined inclusion and exclusion criteria (Fig. 1). Inclusion criteria were unilateral invasive ductal breast cancer diagnosed between 2004 and 2020, age older than 18, tumor size ≥ 5 mm. Exclusion criteria (Fig. 1) were, history of any prior cancer, male gender, metastatic disease at time of diagnosis, tumor size smaller than 5 mm, and neoadjuvant chemotherapy treatment. All cases were reviewed by a clinician to ensure that inclusion and exclusion criteria were met.

The final study population, assembled from the NYPOG institutions, consisted of 418 patients who met the inclusion and exclusion criteria. Patient tissue was stained using IHC triple staining for TMEM doorways (Fig. 1).

Although in 2010 the criterion for calling a case ER+ changed from 10% to 1%, 97% of patients with ER+ disease have more than 10% of cancer cells positive for ER^31,32^. Therefore, subtyping cancer into the ER+ category has not changed for most patients. Additionally, quantitative ER% data were not uniformly available across institutions and were therefore not included in this analysis. Detailed data on specific chemotherapy regimens, endocrine therapy duration, and adherence were not consistently available across all institutions and were therefore not included in the analysis. Treatment was included as a binary covariate (endocrine therapy vs. chemoendocrine therapy) for ER + /HER2− patients.

### TMEM doorway staining

TMEM doorway triple immunostaining was performed as per a previously validated protocol^20,21^, which entails identifying Mena-overexpressing tumor cells with an antibody that stains all isoforms of Mena (anti-pan-Mena antibody, P/N: 610692, BD Biosciences, San Jose, CA, USA, Fast Red chromogen, Bond Polymer Refine Red Detection, Leica Biosystems, Buffalo Grove, IL, USA), macrophages (CD-68 antibody, clone PG-M1; 1:300 dilution; DAKO with antigen retrieval using Bond Epitope Retrieval Solution 1 and 3,3’-Diaminobenzidine (DAB) chromogen), and endothelial cells (CD-31 antibody, clone JC70A; 1:800 dilution; DAKO, Santa Clara, CA, USA with Bond Epitope Retrieval Solution 2 and Vector Blue chromogen).

### TMEM doorway score and quantification

Following staining, slides were digitally scanned at 20x (Pannoramic P250, 3DHistech) and digital slides were imported into the Visiopharm image analysis software (Visiopharm, Hørsholm, Denmark). In Visiopharm, two pathologists (BKF and MO) circled regions of interest that included all tumor areas meeting appropriate pathological criteria such as tumor adequacy, absence of necrosis or inflammation, and lack of retraction and tissue fold artifacts. The total ROI area in each digital whole slide was equivalent to ten high-power (20x) microscope fields (600 × 880 μm^2^), consistent with previous studies^9,21,33,34^.

To identify structures such as blood vessels, macrophages, tumor, and stroma, a linear Bayesian classifier was trained using the manually annotated pixel labels shown in Supplementary Fig. 2. TMEM doorways were then identified and quantified using a previously published algorithm^34^ across entire ROIs in each digital whole slide. TMEM doorway score was defined as the density of TMEM doorways (number of TMEM doorways per square millimeter). The TMEM doorway score high-low cut-off was adapted from a prior study (Rohan et al.) that established the threshold for significant increased risk of distant recurrence^20^. The original Rohan et al. method for calculating the TMEM doorway score involved manually counting the total number of TMEM doorways observed within 10 high-powered fields. However, since we utilized TMEM doorway density in the present study, we converted the Rohan cut point value to its density equivalent by scoring the prior Rohan et al. cohort using the density approach and then using a linear regression (Supplementary Fig. 3) to determine the conversion factor, to ensure consistency with the prior work. Based on this, we classified TMEM doorway scores as low (<15) and high (≥15)^20^. Representative images of tumors with low and high TMEM doorway scores are shown in Supplementary Fig. 4.

### Quantification of macrophage and microvascular densities

TMEM doorway identification and quantification includes classification of pixels into tumor cells, macrophages, and blood vessels across the entire ROI. To quantify macrophage and vessel density, we utilized the macrophage and vessel identification portions of the TMEM doorway algorithm. Macrophage density was calculated by dividing the total macrophage area by the ROI area. Similarly, vascular density was determined by dividing the total vessel area by the ROI area. Representative images of macrophage and microvascular density quantification are shown in Supplementary Fig. 5.

### Sample size and power calculation

With 184 Black patients, 145 White patients, and a total of 158 patients with distant recurrence, this study had 80% power to detect a OR of 1.90 among black patients and 2.00 among white patients when evaluating a binary TMEM doorway score (dichotomized at median) using a two-sided type I error rate of 0.05.

### Statistical Analysis

Patient and tumor characteristics – including age (years); race (Black, White, Asian, other); surgery type (lumpectomy/breast conserving therapy vs mastectomy); tumor size (>5 cm, 2–5 cm, <2 cm); nodal status (positive vs. negative); Nottingham histologic grade (1, 2, 3); tumor subtype (TNBC vs ER + /HER2- vs HER2 + ) – and tumor microenvironment markers – including TMEM doorway score; macrophage density; and microvascular density – were compared between Black and White patients using Wilcoxon rank sum tests for continuous variables and chi-squared tests or Fisher’s exact tests for categorical variables.

The primary outcome variable was development of distant recurrence. Death prior to a distant recurrence or second primary cancer was censored. Univariate logistic regression analysis was used to compare distant metastatic outcome between racial groups and categorized pro-metastatic tumor characteristics (e.g., high vs low TMEM doorway score). An unconditional multivariable logistic regression model was used to examine the association between TMEM doorway score and distant recurrence, adjusting for race, age, BMI, tumor size, nodal status, tumor grade, and treatment.

Statistical significance was specified a priori as *p* < 0.05 and two-side sided p-values were reported. All analyses were conducted using RStudio version 2024.04.2 + 756 and GraphPad Prism version 10.3.1 (Dotmatics, Boston, MA).

## Supplementary information

Racial Disparity_Supplementary Figure v1

## Data Availability

The data generated in this study are available upon request from the corresponding author.
