# Supplementary material for: Racial disparity in pro-metastatic tumor microenvironment in treatment naïve breast cancer
Source: NPJ Breast Cancer. 2026 Jan 6;12:3. doi: 10.1038/s41523-025-00865-1 (PMC12780041; doi:10.1038/s41523-025-00865-1)
Supplement: Supplementary file 1 — Racial Disparity_Supplementary Figure v1 [file 41523_2025_865_MOESM1_ESM.pptx]

## Slide 1
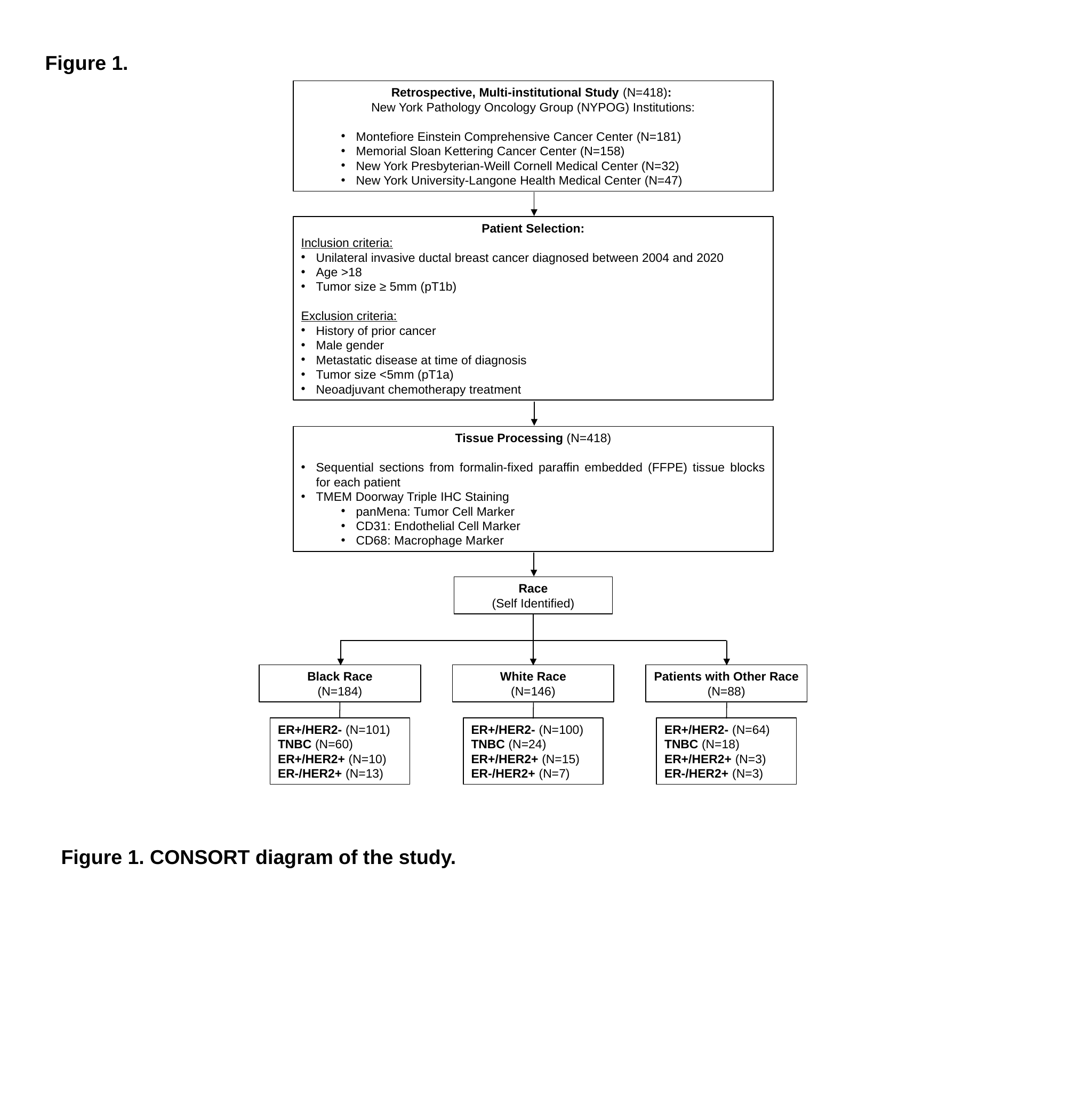

Figure 1.
Retrospective, Multi-institutional Study (N=418):
New York Pathology Oncology Group (NYPOG) Institutions:
Montefiore Einstein Comprehensive Cancer Center (N=181)
Memorial Sloan Kettering Cancer Center (N=158)
New York Presbyterian-Weill Cornell Medical Center (N=32)
New York University-Langone Health Medical Center (N=47)
Patient Selection:
Inclusion criteria:
Unilateral invasive ductal breast cancer diagnosed between 2004 and 2020
Age >18
Tumor size ≥ 5mm (pT1b)
Exclusion criteria:
History of prior cancer
Male gender
Metastatic disease at time of diagnosis
Tumor size <5mm (pT1a)
Neoadjuvant chemotherapy treatment
Tissue Processing (N=418)
Sequential sections from formalin-fixed paraffin embedded (FFPE) tissue blocks for each patient
TMEM Doorway Triple IHC Staining
panMena: Tumor Cell Marker
CD31: Endothelial Cell Marker
CD68: Macrophage Marker
Race
(Self Identified)
Black Race
(N=184)
White Race
(N=146)
Patients with Other Race
(N=88)
ER+/HER2- (N=101)
TNBC (N=60)
ER+/HER2+ (N=10)
ER-/HER2+ (N=13)
ER+/HER2- (N=100)
TNBC (N=24)
ER+/HER2+ (N=15)
ER-/HER2+ (N=7)
ER+/HER2- (N=64)
TNBC (N=18)
ER+/HER2+ (N=3)
ER-/HER2+ (N=3)
Figure 1. CONSORT diagram of the study.

## Slide 2
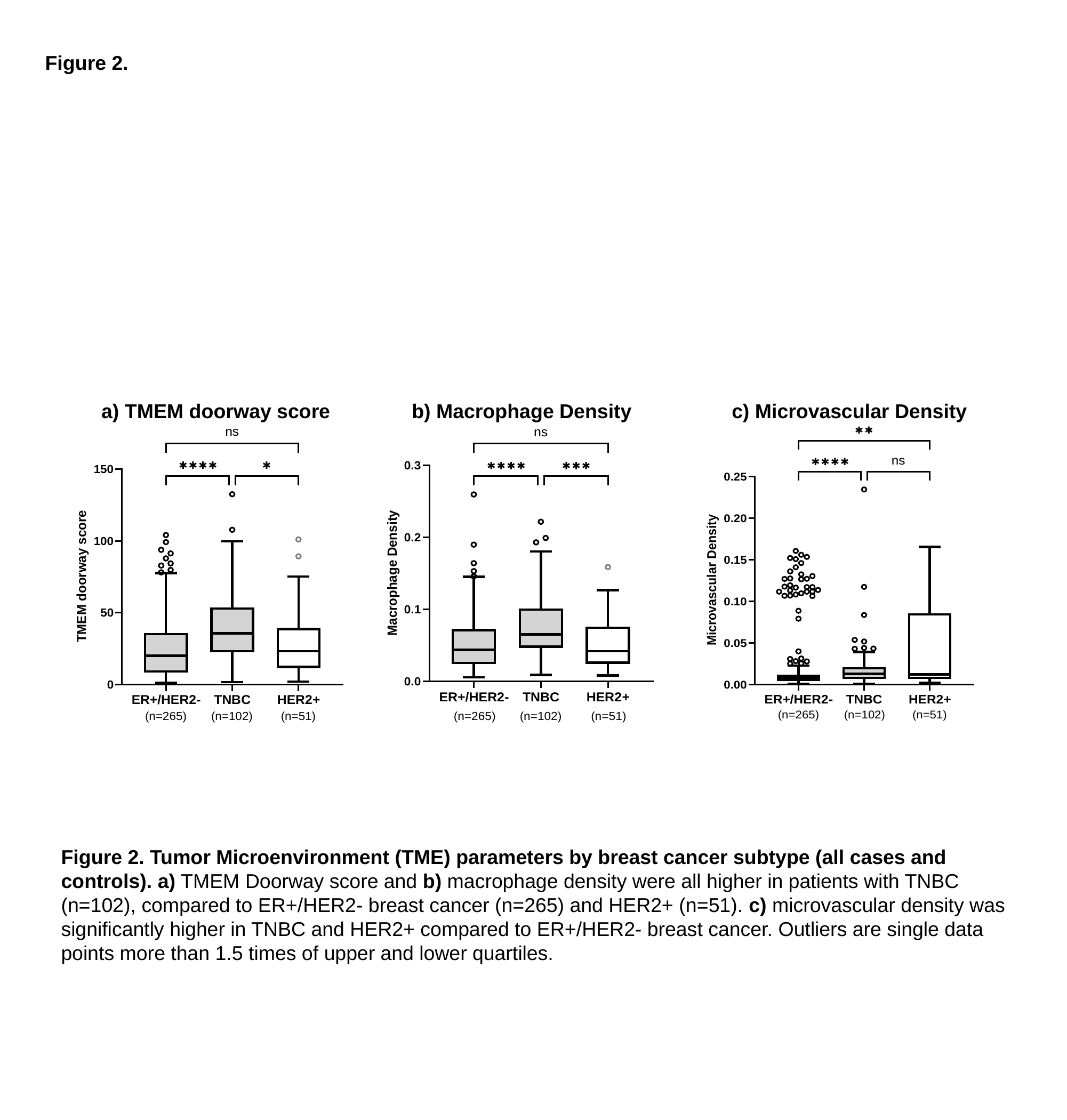

Figure 2.
a) TMEM doorway score
b) Macrophage Density
c) Microvascular Density
Figure 2. Tumor Microenvironment (TME) parameters by breast cancer subtype (all cases and controls). a) TMEM Doorway score and b) macrophage density were all higher in patients with TNBC (n=102), compared to ER+/HER2- breast cancer (n=265) and HER2+ (n=51). c) microvascular density was significantly higher in TNBC and HER2+ compared to ER+/HER2- breast cancer. Outliers are single data points more than 1.5 times of upper and lower quartiles.

## Slide 3
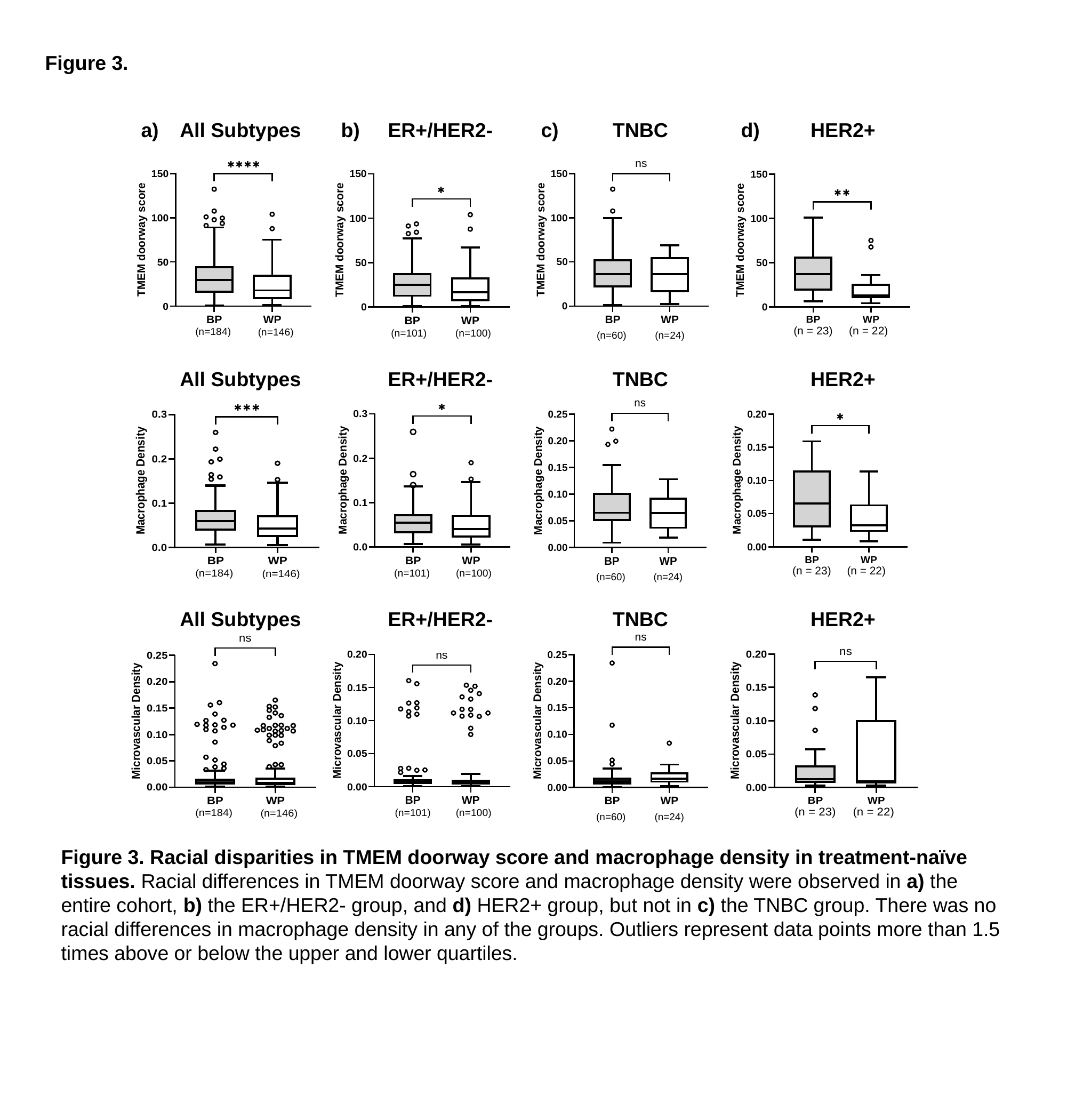

Figure 3.
a)
b)
c)
d)
All Subtypes
ER+/HER2-
TNBC
HER2+
All Subtypes
ER+/HER2-
TNBC
HER2+
All Subtypes
ER+/HER2-
TNBC
HER2+
Figure 3. Racial disparities in TMEM doorway score and macrophage density in treatment-naïve tissues. Racial differences in TMEM doorway score and macrophage density were observed in a) the entire cohort, b) the ER+/HER2- group, and d) HER2+ group, but not in c) the TNBC group. There was no racial differences in macrophage density in any of the groups. Outliers represent data points more than 1.5 times above or below the upper and lower quartiles.

## Slide 4
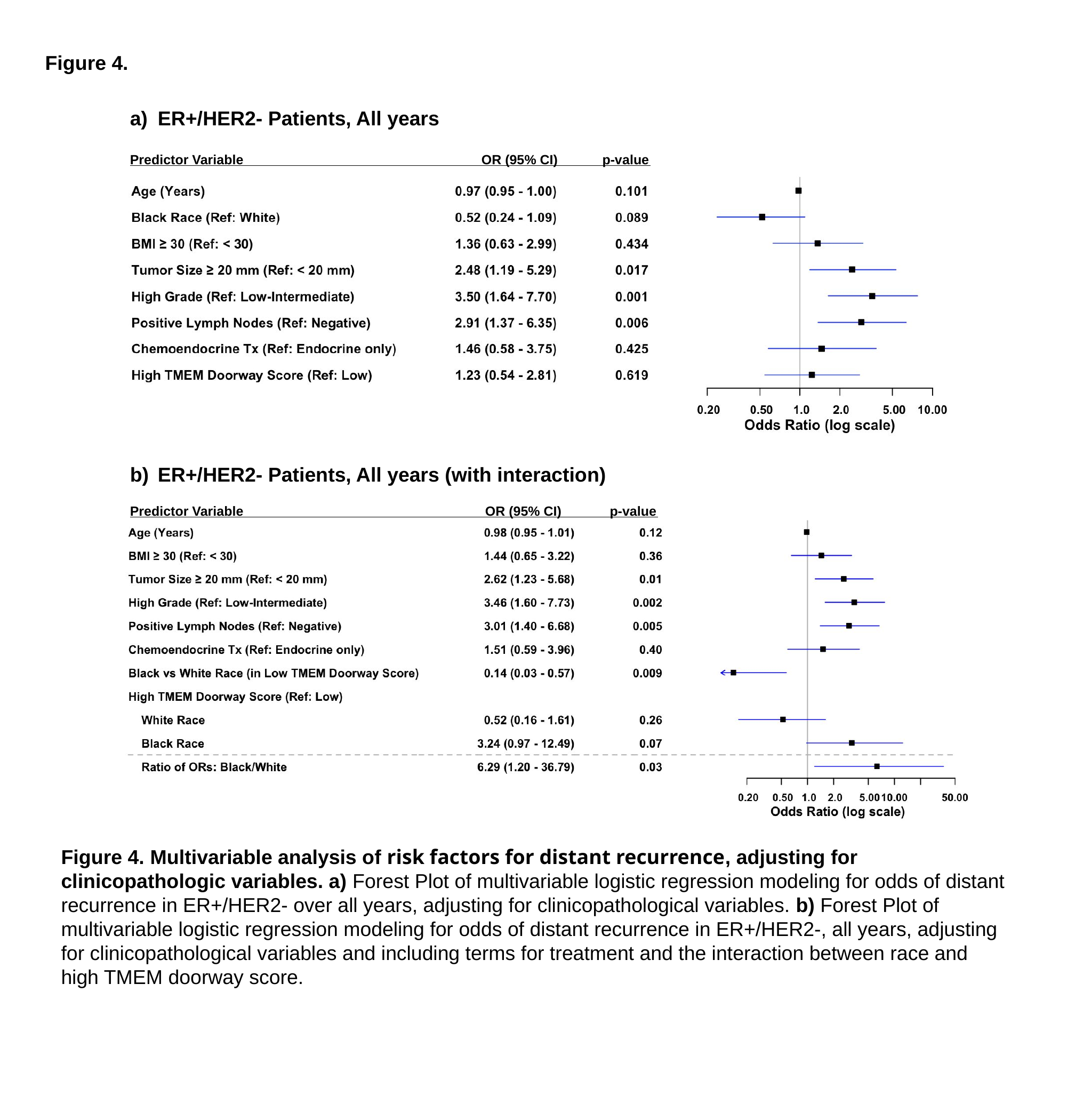

Figure 4.
a)
ER+/HER2- Patients, All years
Predictor Variable OR (95% CI) p-value
b)
ER+/HER2- Patients, All years (with interaction)
Predictor Variable OR (95% CI) p-value
Figure 4. Multivariable analysis of risk factors for distant recurrence, adjusting for clinicopathologic variables. a) Forest Plot of multivariable logistic regression modeling for odds of distant recurrence in ER+/HER2- over all years, adjusting for clinicopathological variables. b) Forest Plot of multivariable logistic regression modeling for odds of distant recurrence in ER+/HER2-, all years, adjusting for clinicopathological variables and including terms for treatment and the interaction between race and high TMEM doorway score.

## Slide 5
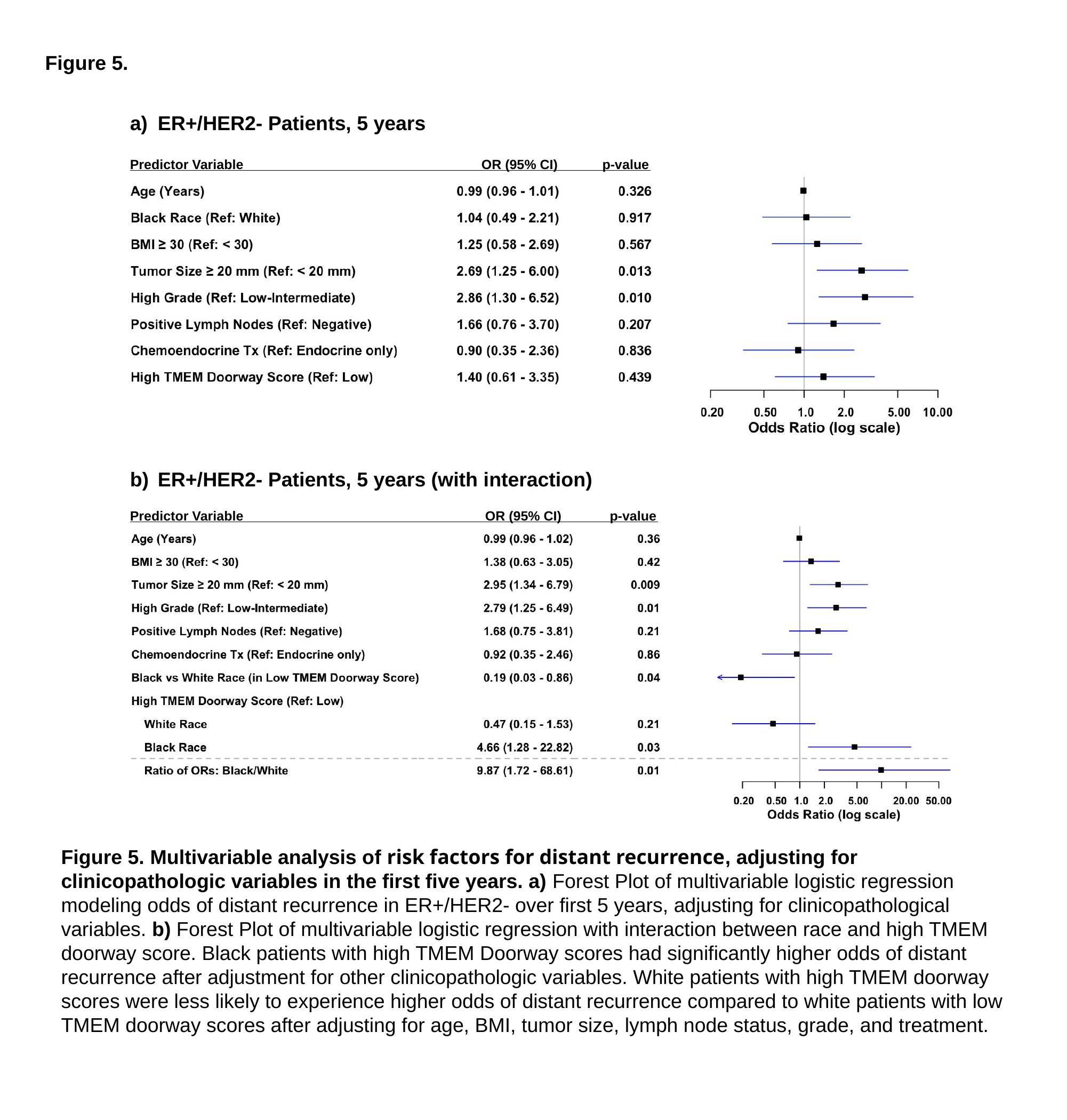

Figure 5.
a)
ER+/HER2- Patients, 5 years
Predictor Variable OR (95% CI) p-value
b)
ER+/HER2- Patients, 5 years (with interaction)
Predictor Variable OR (95% CI) p-value
Figure 5. Multivariable analysis of risk factors for distant recurrence, adjusting for clinicopathologic variables in the first five years. a) Forest Plot of multivariable logistic regression modeling odds of distant recurrence in ER+/HER2- over first 5 years, adjusting for clinicopathological variables. b) Forest Plot of multivariable logistic regression with interaction between race and high TMEM doorway score. Black patients with high TMEM Doorway scores had significantly higher odds of distant recurrence after adjustment for other clinicopathologic variables. White patients with high TMEM doorway scores were less likely to experience higher odds of distant recurrence compared to white patients with low TMEM doorway scores after adjusting for age, BMI, tumor size, lymph node status, grade, and treatment.

## Slide 6
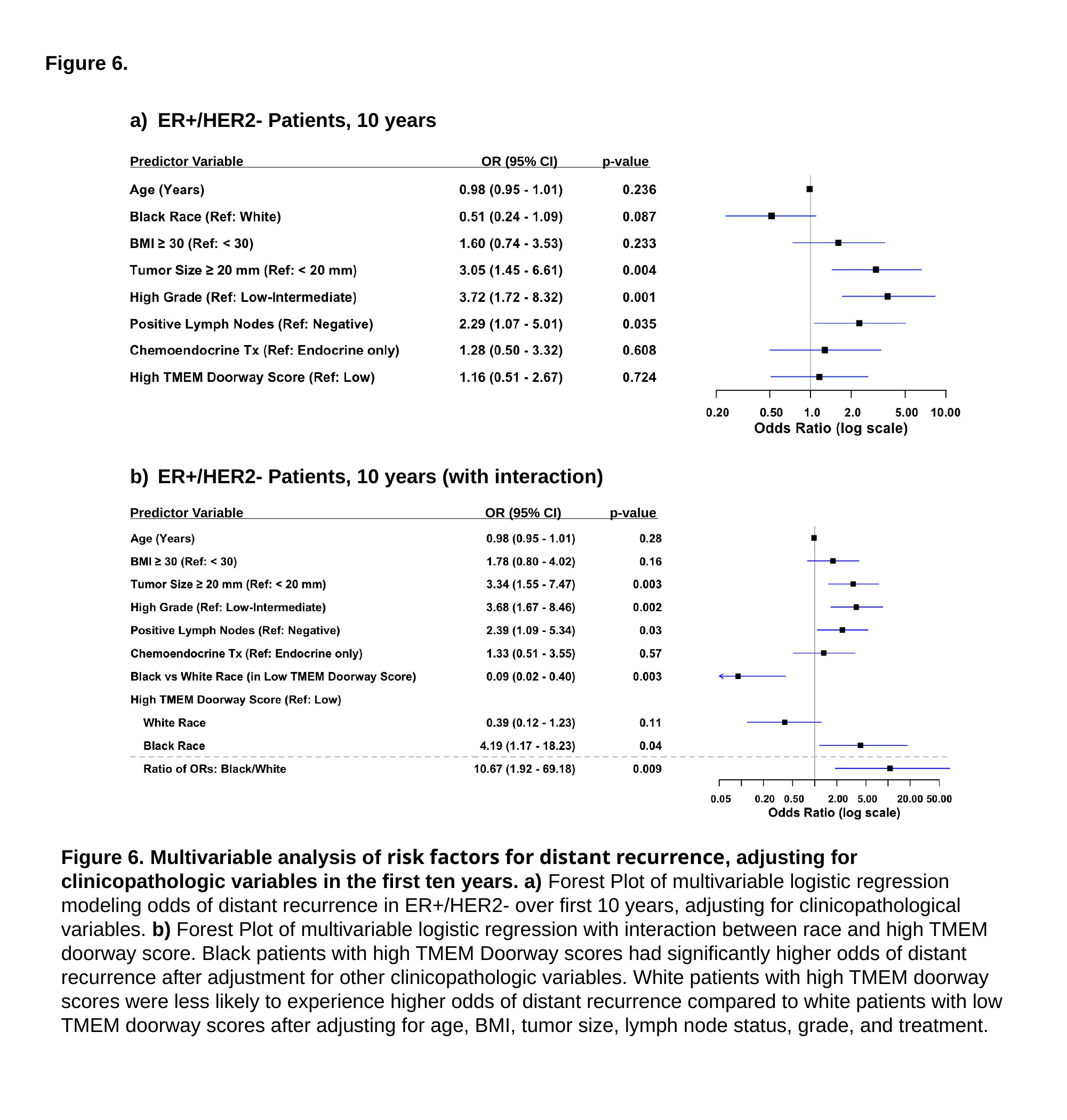

Figure 6.
a)
ER+/HER2- Patients, 10 years
Predictor Variable OR (95% CI) p-value
b)
ER+/HER2- Patients, 10 years (with interaction)
Predictor Variable OR (95% CI) p-value
Figure 6. Multivariable analysis of risk factors for distant recurrence, adjusting for clinicopathologic variables in the first ten years. a) Forest Plot of multivariable logistic regression modeling odds of distant recurrence in ER+/HER2- over first 10 years, adjusting for clinicopathological variables. b) Forest Plot of multivariable logistic regression with interaction between race and high TMEM doorway score. Black patients with high TMEM Doorway scores had significantly higher odds of distant recurrence after adjustment for other clinicopathologic variables. White patients with high TMEM doorway scores were less likely to experience higher odds of distant recurrence compared to white patients with low TMEM doorway scores after adjusting for age, BMI, tumor size, lymph node status, grade, and treatment.

## Slide 7
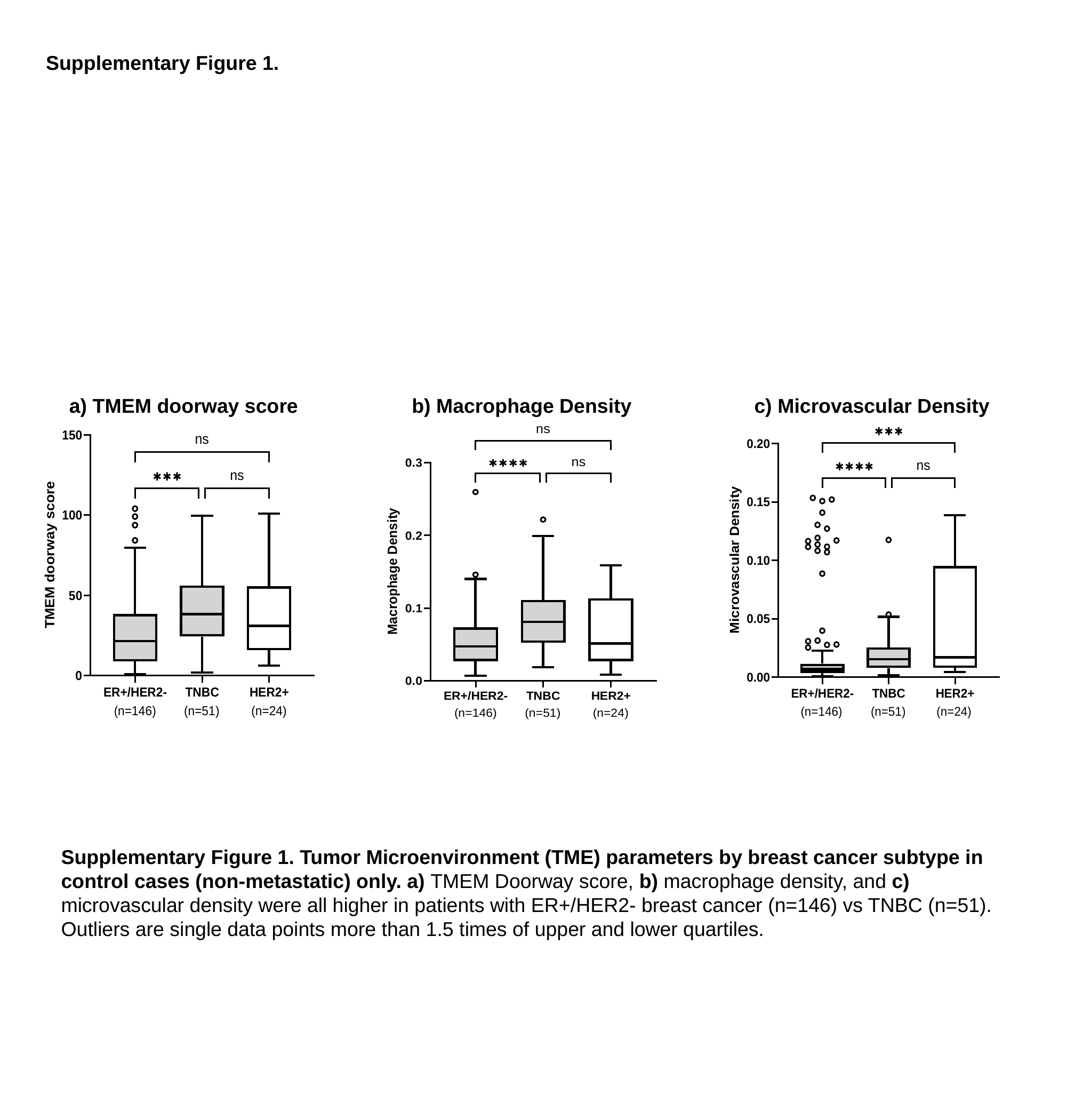

Supplementary Figure 1.
a) TMEM doorway score
b) Macrophage Density
c) Microvascular Density
Supplementary Figure 1. Tumor Microenvironment (TME) parameters by breast cancer subtype in control cases (non-metastatic) only. a) TMEM Doorway score, b) macrophage density, and c) microvascular density were all higher in patients with ER+/HER2- breast cancer (n=146) vs TNBC (n=51). Outliers are single data points more than 1.5 times of upper and lower quartiles.

## Slide 8
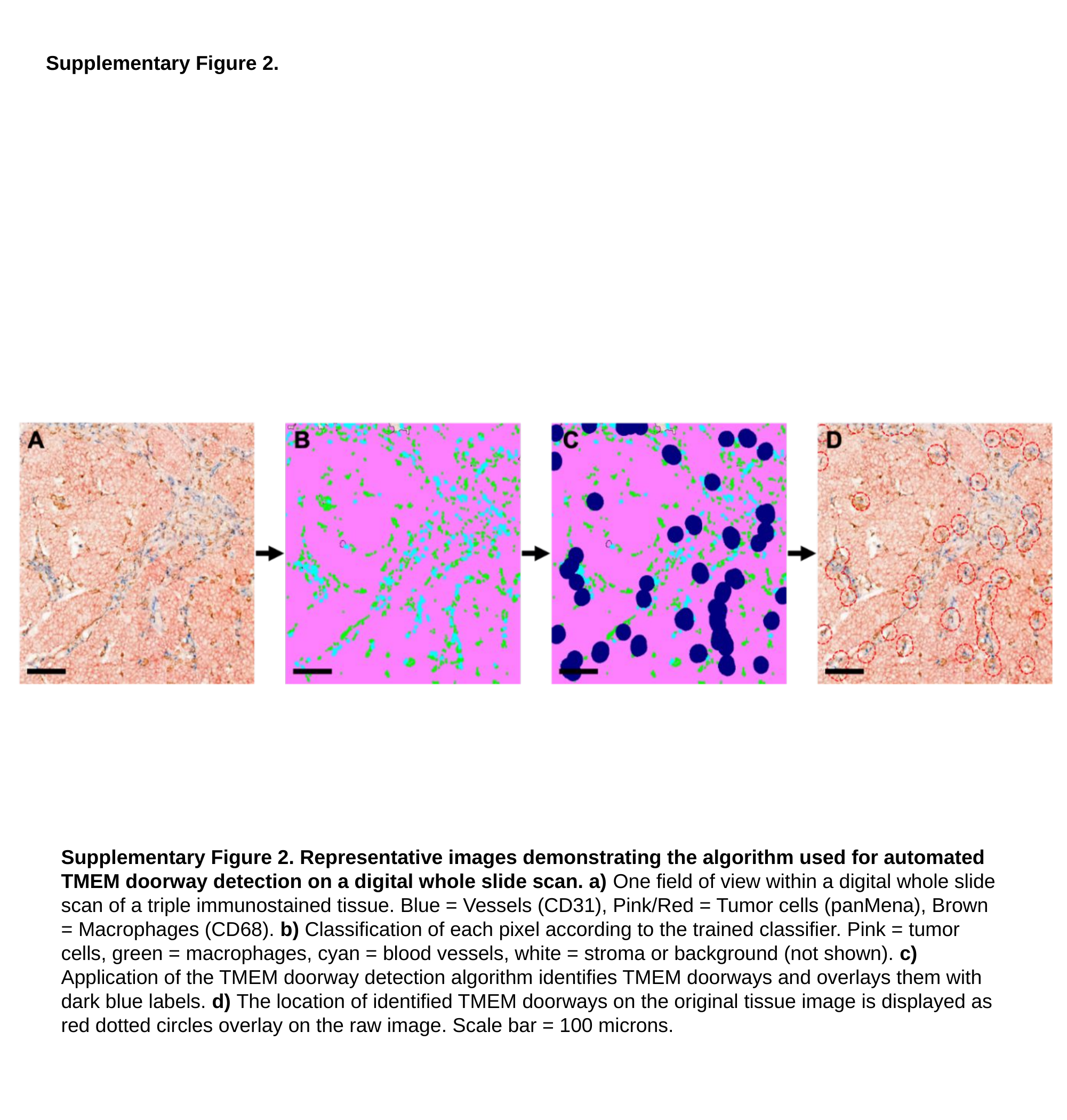

Supplementary Figure 2.
Supplementary Figure 2. Representative images demonstrating the algorithm used for automated TMEM doorway detection on a digital whole slide scan. a) One field of view within a digital whole slide scan of a triple immunostained tissue. Blue = Vessels (CD31), Pink/Red = Tumor cells (panMena), Brown = Macrophages (CD68). b) Classification of each pixel according to the trained classifier. Pink = tumor cells, green = macrophages, cyan = blood vessels, white = stroma or background (not shown). c) Application of the TMEM doorway detection algorithm identifies TMEM doorways and overlays them with dark blue labels. d) The location of identified TMEM doorways on the original tissue image is displayed as red dotted circles overlay on the raw image. Scale bar = 100 microns.

## Slide 9
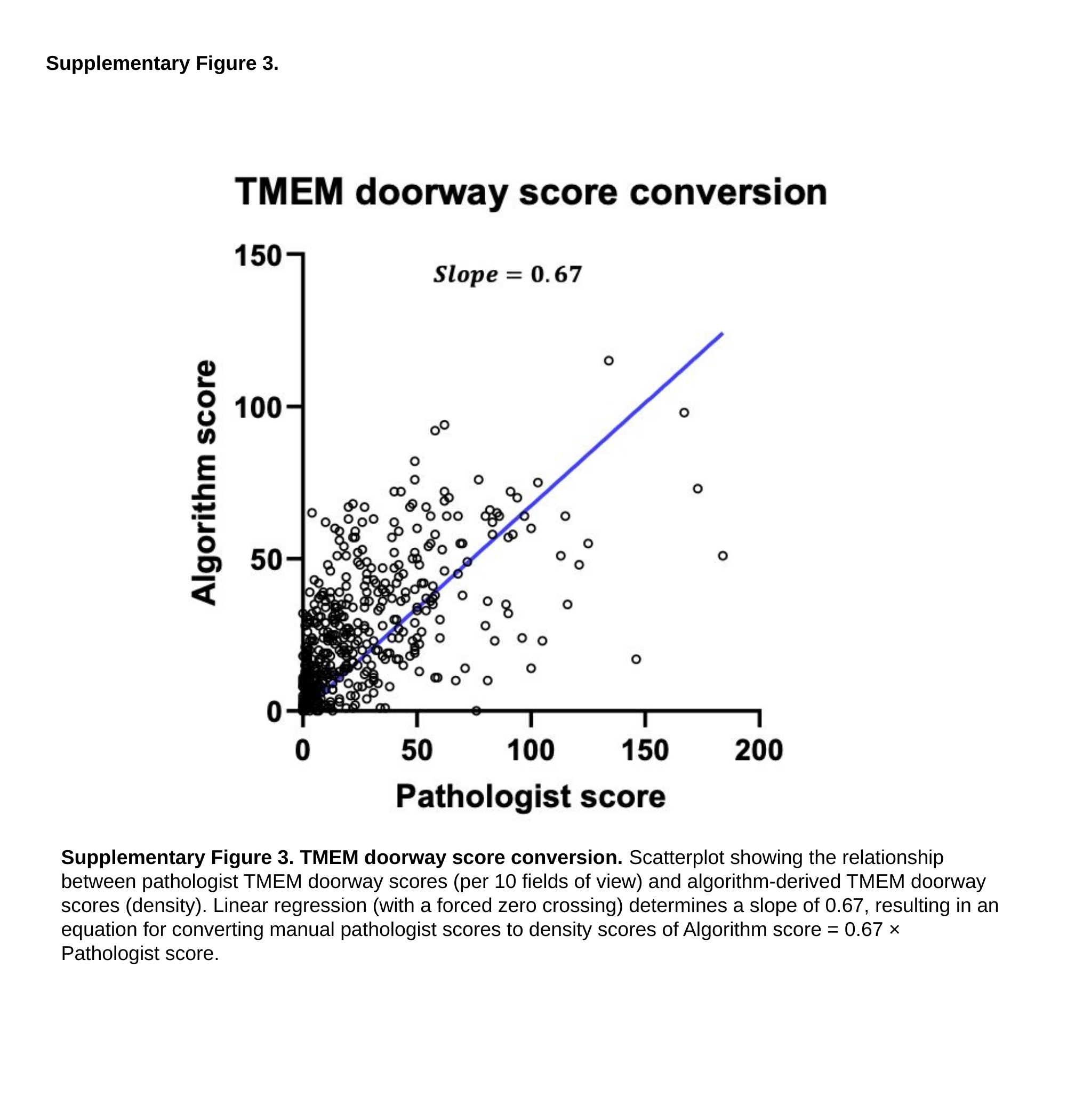

Supplementary Figure 3.
Supplementary Figure 3. TMEM doorway score conversion. Scatterplot showing the relationship between pathologist TMEM doorway scores (per 10 fields of view) and algorithm-derived TMEM doorway scores (density). Linear regression (with a forced zero crossing) determines a slope of 0.67, resulting in an equation for converting manual pathologist scores to density scores of Algorithm score = 0.67 × Pathologist score.

## Slide 10
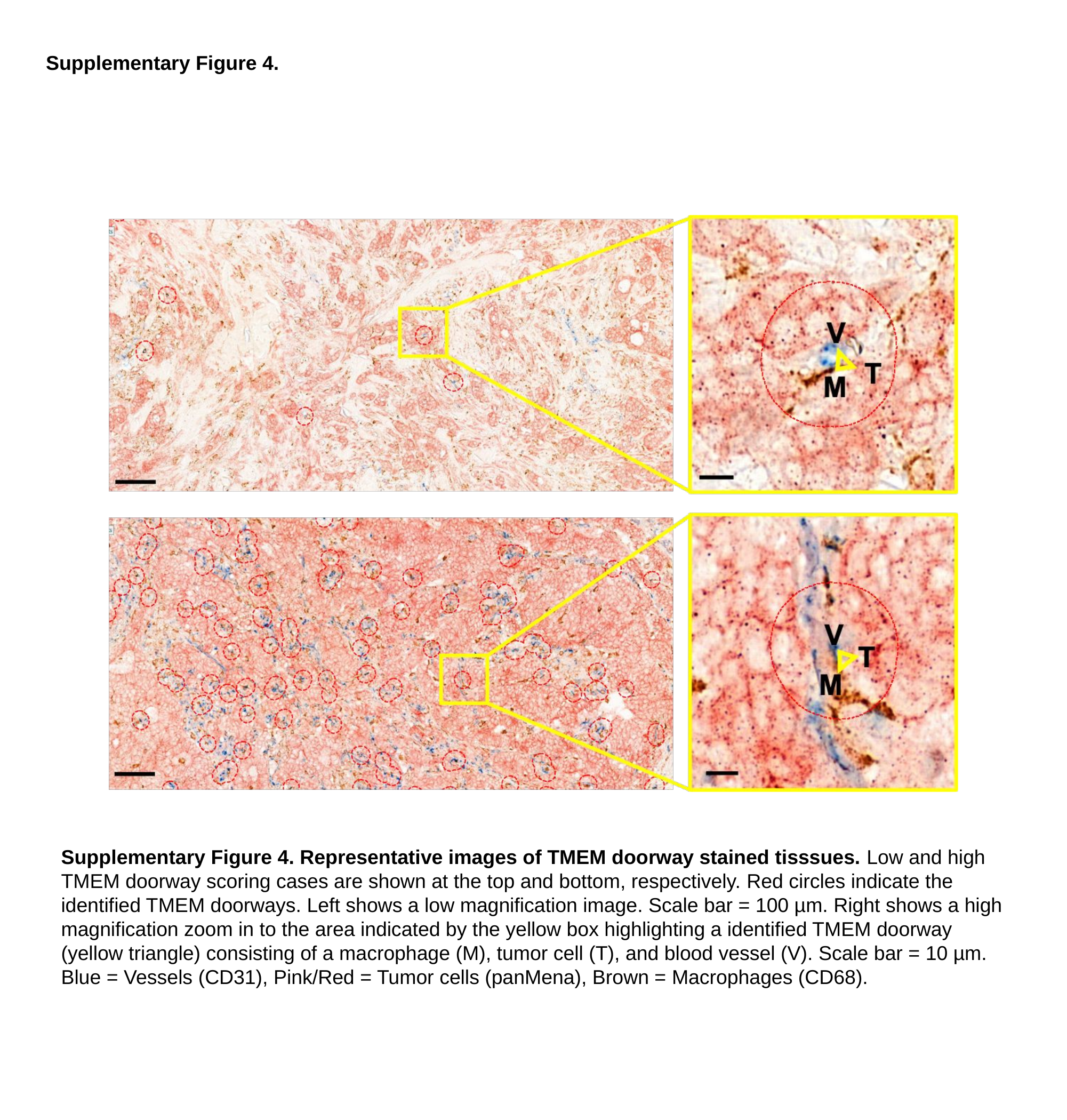

Supplementary Figure 4.
Supplementary Figure 4. Representative images of TMEM doorway stained tisssues. Low and high TMEM doorway scoring cases are shown at the top and bottom, respectively. Red circles indicate the identified TMEM doorways. Left shows a low magnification image. Scale bar = 100 µm. Right shows a high magnification zoom in to the area indicated by the yellow box highlighting a identified TMEM doorway (yellow triangle) consisting of a macrophage (M), tumor cell (T), and blood vessel (V). Scale bar = 10 µm. Blue = Vessels (CD31), Pink/Red = Tumor cells (panMena), Brown = Macrophages (CD68).

## Slide 11
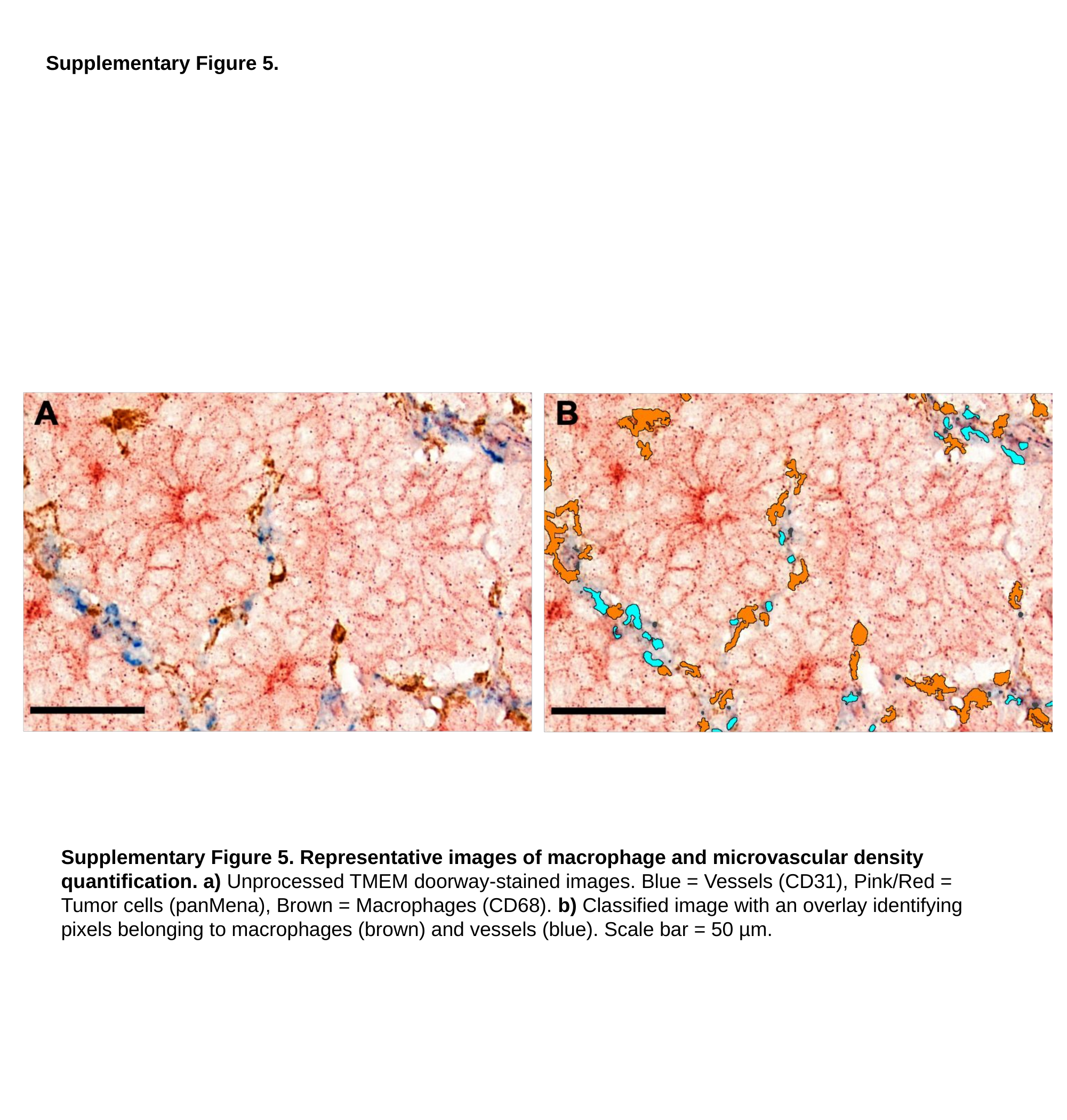

Supplementary Figure 5.
Supplementary Figure 5. Representative images of macrophage and microvascular density quantification. a) Unprocessed TMEM doorway-stained images. Blue = Vessels (CD31), Pink/Red = Tumor cells (panMena), Brown = Macrophages (CD68). b) Classified image with an overlay identifying pixels belonging to macrophages (brown) and vessels (blue). Scale bar = 50 µm.

## Slide 12
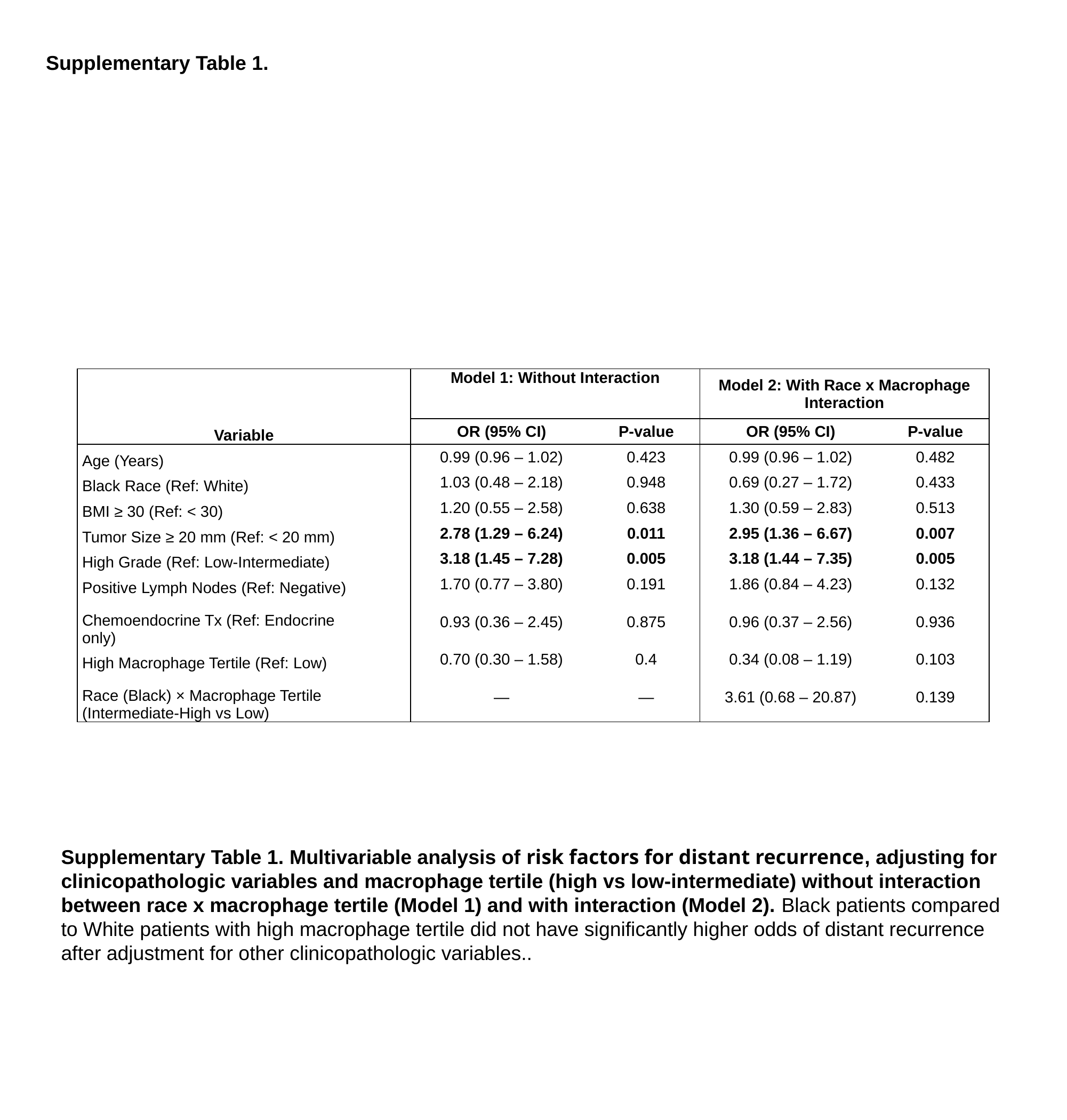

Supplementary Table 1.
| | Model 1: Without Interaction | | Model 2: With Race x Macrophage Interaction | |
| --- | --- | --- | --- | --- |
| Variable | OR (95% CI) | P-value | OR (95% CI) | P-value |
| Age (Years) | 0.99 (0.96 – 1.02) | 0.423 | 0.99 (0.96 – 1.02) | 0.482 |
| Black Race (Ref: White) | 1.03 (0.48 – 2.18) | 0.948 | 0.69 (0.27 – 1.72) | 0.433 |
| BMI ≥ 30 (Ref: < 30) | 1.20 (0.55 – 2.58) | 0.638 | 1.30 (0.59 – 2.83) | 0.513 |
| Tumor Size ≥ 20 mm (Ref: < 20 mm) | 2.78 (1.29 – 6.24) | 0.011 | 2.95 (1.36 – 6.67) | 0.007 |
| High Grade (Ref: Low-Intermediate) | 3.18 (1.45 – 7.28) | 0.005 | 3.18 (1.44 – 7.35) | 0.005 |
| Positive Lymph Nodes (Ref: Negative) | 1.70 (0.77 – 3.80) | 0.191 | 1.86 (0.84 – 4.23) | 0.132 |
| Chemoendocrine Tx (Ref: Endocrine only) | 0.93 (0.36 – 2.45) | 0.875 | 0.96 (0.37 – 2.56) | 0.936 |
| High Macrophage Tertile (Ref: Low) | 0.70 (0.30 – 1.58) | 0.4 | 0.34 (0.08 – 1.19) | 0.103 |
| Race (Black) × Macrophage Tertile (Intermediate-High vs Low) | — | — | 3.61 (0.68 – 20.87) | 0.139 |
Supplementary Table 1. Multivariable analysis of risk factors for distant recurrence, adjusting for clinicopathologic variables and macrophage tertile (high vs low-intermediate) without interaction between race x macrophage tertile (Model 1) and with interaction (Model 2). Black patients compared to White patients with high macrophage tertile did not have significantly higher odds of distant recurrence after adjustment for other clinicopathologic variables..
